# The role of nutritional vitamin D on microinflammation and nutritional status in maintenance hemodialysis patients: a meta-analysis of randomized controlled trials

**DOI:** 10.3389/fnut.2026.1767616

**Published:** 2026-05-29

**Authors:** Chen Li, Shuangshuang Xu, Yu Wang, Meiyan Wu

**Affiliations:** Department of Nephrology, The First Hospital of Jilin University, Jilin University, Changchun, China

**Keywords:** maintenance hemodialysis, meta-analysis, microinflammation, nutritional vitamin D, RCT

## Abstract

**Background:**

Microinflammation is widespread in Maintenance hemodialysis (MHD) patients. The continuous interaction of microinflammation and malnutrition is an independent risk factor for the clinical prognosis of MHD patients. Nutritional vitamin D deficiency is closely related to chronic kidney disease inflammation, but whether correcting this deficiency can improve microinflammation in the MHD population remains unclear.

**Methods:**

Studies published up to March 1, 2025 were included. Risk of bias was assessed using the Cochrane tool. RevMan 5.4 and Stata 17 were used for analyses, with heterogeneity assessed and random-effects models applied. Key outcomes included CRP, serum albumin, TNF-α, IL-1β, immunoreactive Parathyroid Hormone (iPTH), adverse events, hypercalcemia, and hyperphosphatemia.

**Results:**

A total of 617 patients in eight randomized controlled trials were included. The experimental group interventions were nutritional vitamin D, while those of the control group were placebo. The meta-analysis showed that the decrease in CRP in the nutritional vitamin D intervention group was significantly higher than that in the control group [MD −3.15, 95% confidence interval (CI; −4.46, −1.84), *P* < 0.05]. Subgroup analysis demonstrated that nutritional vitamin D supplementation not only reduced CRP in MHD patients with baseline vitamin D deficiency, but also in those with normal vitamin D baseline levels [MD −2.64, 95% CI (−4.47, −0.81), *P* < 0.05; MD −3.97, 95% CI (−5.78, −2.17), *P* < 0.05]. The increase in serum albumin in the vitamin D group was more significant than in the control group [MD 0.55, 95% CI (0.10, 1.01), *P* < 0.05]. iPTH decreased more significantly in the vitamin D group than in the control group [SMD −0.56, 95% CI (−1.07, −0.05), *P* < 0.05]. In terms of safety, the overall incidence of adverse reactions in the nutritional vitamin D group was similar to that in the control group (*P* = 0.09). Moreover, no significant differences were found in the incidence of hypercalcemia and hyperphosphatemia between the two groups (*P* = 0.15, *P* = 0.32).

**Conclusion:**

Nutritional vitamin D supplementation may improve microinflammation and nutritional status in MHD patients, with potential benefits for bone metabolism and no apparent increase in the risk of hypercalcemia or hyperphosphatemia.

## 1 Introduction

The number of patients with end-stage renal disease (ESRD) and those undergoing maintenance hemodialysis (MHD) is increasing annually (1). According to the statistics of the Chinese National Renal Data System platform, by the end of December 2024, the number of hemodialysis patients in China had exceeded one million, indicating a rapid growth trend. 2024 annual report of the United States Renal Data System (USRDS) exhibited that the mortality of patients undergoing hemodialysis in 2022 was significantly higher than in 2012 (2). Therefore, the survival prognosis of MHD patients have become a global public health problem that should not be ignored.

Microinflammation is defined as the absence of systemic or local dominant clinical signs of infection; however, it is characterized by a low-level, persistent inflammatory state, manifested by a slight increase in C-reactive protein (CRP) and inflammatory factors. The persistence of the microinflammatory state in MHD can aggravate the occurrence of cardio-vascular events by worsening coronary artery calcification (3), which is an independent risk factor for cardiovascular events and the main cause of death in the MHD population. At the same time, microinflammation can induce or aggravate malnutrition by enhancing the body's catabolism and reducing the synthesis of the serum albumin (4). Inflammation–malnutrition–atherosclerosis syndrome is formed by the interaction of microinflammation, malnutrition, and atherosclerosis. It is a key factor affecting the long-term survival rate and prognosis of MHD patients. However, it still remains difficult to effectively prevent the occurrence and progression of microinflammation–malnutrition in MHD patients.

Vitamin D is uncovered that closely related to inflammation in chronic kidney disease (CKD) patients. Vitamin D acts on the signal transduction pathway through autocrine or paracrine signaling mediated by the nuclear vitamin D receptor and plays an immunomodulatory role. Vitamin D deficiency inhibits macrophage maturation (5). It has been reported that Vitamin D could modulate inflammatory responses through pathways such as nuclear factor kappa-light-chain-enhancer of activated B cells (NF-κB), and reduce the production of pro-inflammatory mediators such as tumor necrosis factor-alpha (TNF-α) and CRP (6, 7). Vitamin D can also reduce the secretion of TNF-α by human monocytes/macrophages through various mechanisms, consequently reducing inflammation (8–10).

Vitamin D insufficiency and deficiency are common in the MHD population (11). Previous evidence has suggested a potential association between vitamin D status and micro-inflammatory state in hemodialysis patients (12). However, the available evidence remains limited, and the effects of nutritional vitamin D supplementation on microinflammation, nutritional status, CKD-mineral bone disease (MBD)-related outcomes, and safety in MHD patients have not been systematically evaluated based on randomized controlled trials. Therefore, the present study aimed to systematically review and meta-analyze randomized controlled trials to determine whether, in maintenance hemodialysis patients, nutritional vitamin D supplementation, compared with placebo, can improve microinflammation, nutritional status, and CKD-MBD-related outcomes, and to evaluate its safety.

## 2 Materials and methods

We conducted this study in accordance with the Preferred Reporting Items for Systematic Reviews and Meta-Analyses (PRISMA 2020) statement (13). The completed PRISMA 2020 checklist is provided in the Supplementary File 3. The study protocol was registered with PROSPERO (No. CRD420251002256, publicly accessible at https://www.crd.york.ac.uk/PROSPERO/view/CRD420251002256).

### 2.1 Search strategy

We conducted a comprehensive search of the Medline, Embase, Cochrane Library, and Chinese BioMedical Literature databases from inception to March 1, 2025. The search strategy combined controlled vocabulary terms, where available, and free-text terms related to vitamin D, vitamin D receptor, inflammation, chronic kidney disease, renal dialysis, and hemodialysis. No language restrictions were applied during the literature search. All studies finally included in the review were published in English. The literature search was limited to formal bibliographic databases of published studies; gray literature and trial registries were not systematically searched. This limitation may have introduced potential publication and selection bias, and the findings should therefore be interpreted with caution. The complete Boolean search strategies for each database are provided in Supplementary material 8 in Supplementary File 1.

### 2.2 Selection criteria

Only randomized controlled trials (RCTs) were included. The inclusion criteria were as follows: (1) participants were maintenance hemodialysis patients who had received hemodialysis for at least 3 months; (2) the intervention in the treatment group was nutritional vitamin D supplementation, specifically ergocalciferol and/or cholecalciferol, while the control group received placebo; (3) studies reported at least one inflammation-related outcome, such as CRP, TNF-α, or interleukin-1 beta (IL-1β); and (4) nutritional, bone metabolism, and safety outcomes, including albumin, iPTH, calcium, phosphate, hypercalcemia, or hyperphosphatemia, were additionally extracted when available. The exclusion criteria were as follows: (1) non-randomized studies, observational studies, case reports, reviews, conference abstracts, animal studies, and *in vitro* studies; (2) studies involving peritoneal dialysis patients, kidney transplant recipients, non-dialysis CKD patients, or mixed populations from which data for maintenance hemodialysis patients could not be extracted separately; (3) studies in which the primary intervention was not nutritional vitamin D supplementation, studies using active vitamin D or its analogs as the main intervention; and (4) duplicate publications, multiple reports from the same cohort without additional usable data, or studies with insufficient data for extraction or meta-analysis.

### 2.3 Study selection and data extraction

According to the inclusion criteria and exclusion criteria, the two authors of the meta-analysis independently screened the studies and extracted data using a predesigned standardized data extraction form. During data preparation, unit harmonization, conversion of medians (interquartile ranges) to means (standard deviations) when necessary, and calculation of pre- and post-intervention differences were performed before compilation of the final analytical dataset. After independent extraction, the two reviewers cross-checked the data, and any disagreements were resolved by review from the corresponding author.

### 2.4 Definitions of the outcome measures and treatment effects

The outcomes in this review were classified into four domains according to their clinical focus and biological relevance to nutritional vitamin D supplementation in maintenance hemodialysis patients: inflammation–nutrition-related outcomes, vitamin D-related outcomes, CKD-MBD-related outcomes, and safety outcomes. Inflammation–nutrition-related outcomes were considered the primary outcomes because they directly addressed the main research question, whereas vitamin D-related, CKD-MBD-related, and safety outcomes were considered secondary outcomes, providing additional information on the biological effects, broader metabolic impact, and safety of the intervention. Treatment effects were primarily evaluated as the mean changes from baseline to the end of treatment. For inflammation–nutrition-related outcomes, the main indices included CRP and albumin. For vitamin D-related outcomes, the indices included 25(OH)D and 1,25(OH)_2_D levels. For CKD-MBD-related outcomes, the indices included blood calcium, phosphorus, iPTH, alkaline phosphatase, and FGF-23. Safety outcomes were assessed using treatment-emergent adverse events (TEAEs), including the overall incidence of TEAEs and the incidence of hypercalcemia and hyperphosphatemia. TEAEs were defined as the proportion of participants experiencing at least one adverse reaction during the clinical trial.

### 2.5 Statistical analysis

The evaluation of binary variables was displayed by the relative risk and 95% confidence interval. The continuous variables are represented by the weighted mean differences (WMDs). If the data are expressed in the form of median, quartile, standard error, or 95% CI, they are converted into mean and standard deviation through the conversion formula (14, 15) for the statistical analysis. For data that cannot be unified in units, the standardized mean difference (SMD) is used to eliminate the impact of unit differences. We used Cochran's *Q*-test, the *I*^2^ statistic, and the between-study variance (τ^2^) to assess heterogeneity among the included studies, with *P* < 0.10 or *I*^2^ > 50% considered indicative of substantial heterogeneity. Given the expected clinical and methodological variability across studies, a random-effects model was used as the primary approach for pooled analyses. We additionally performed Hartung–Knapp–Sidik–Jonkman (HKSJ) adjustment as a sensitivity analysis to assess the robustness of the random-effects meta-analysis results. The primary analyses were still based on the conventional random-effects model, while the HKSJ-adjusted results were used as supplementary evidence for robustness assessment. Sensitivity or subgroup analyses were used to explore heterogeneity. The sensitivity analysis was performed by using Stata 17.0 (StataCorp LLC, College Station, TX, USA). The results were deemed stable when the change was small; the direction was the same; the 95% CI overlapped considerably, and the significance remained unchanged (all *P* < 0.05). Meanwhile, a study was considered to significantly affect the results if the combined effect changed substantially after an exclusion or if its 95% CI contained 1 (for OR or RR) or 0 (for WMDs or SMD) or the *P* value > 0.05. Funnel plots and Begg's test were used to assess potential publication bias, where applicable. A *P* value < 0.05 was considered indicative of possible publication bias. Given the limited number of included studies for some outcomes, these analyses were interpreted with caution. Risk of bias was assessed using the Cochrane risk of bias framework in RevMan 5.4.1, with each domain judged as low, unclear, or high risk of bias. This meta-analysis was conducted in strict accordance with the PRISMA guidelines. Review Manager Version 5.4 (Cochrane Center in Northern Europe, Cochrane Collaboration Network, Copenhagen, 2020) was used to generate the forest and funnel maps. Stata 17.0 was used for Begg's test and the subsequent trim-and-fill analysis and sensitivity analyses.

## 3 Results

### 3.1 Summary of the included studies

**Figure 1:**
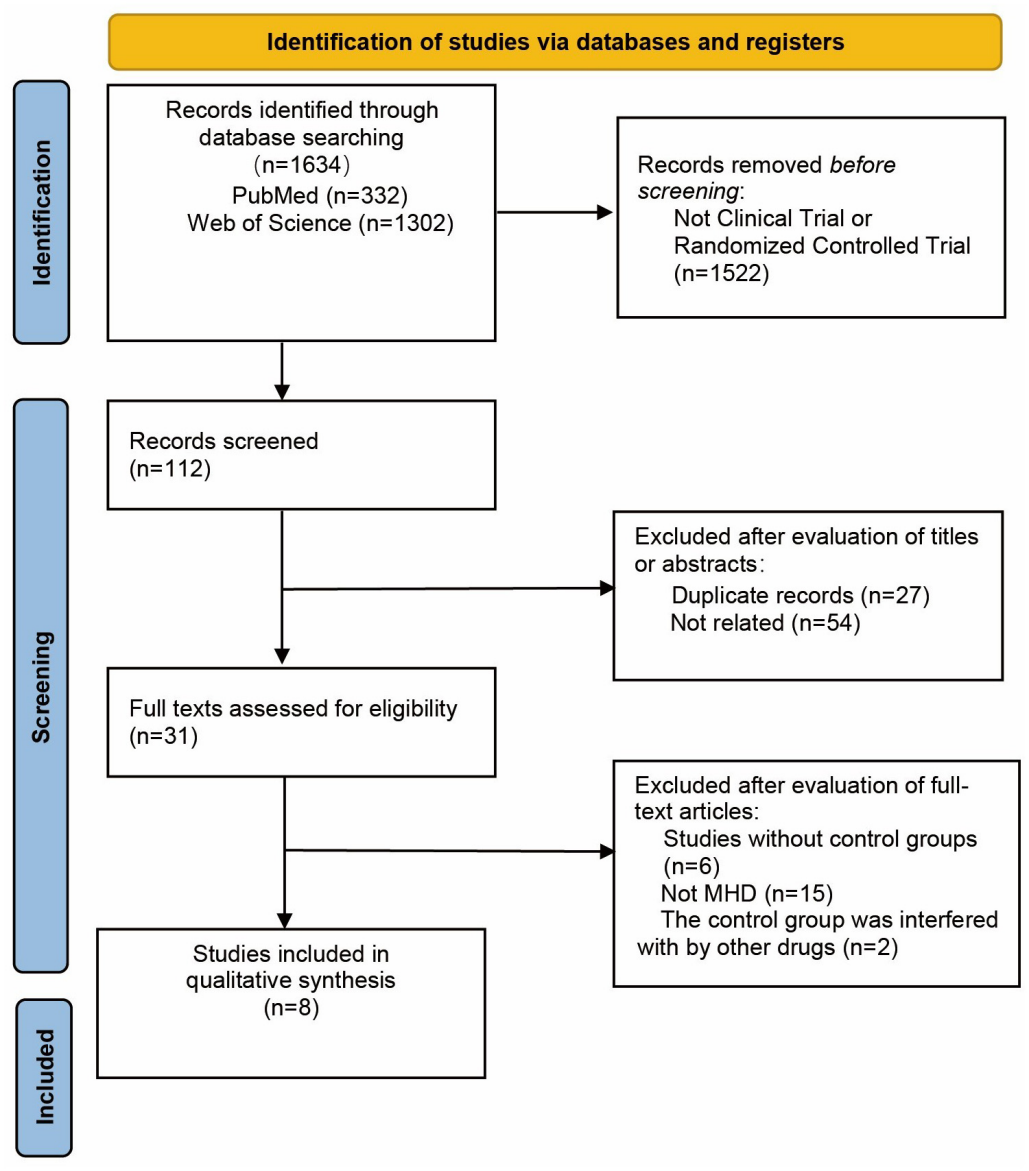
Flowchart illustrating the study selection.

A total of 1,634 records were identified through database searching. After removal of 1,522 records that were not clinical trials or randomized controlled trials, 112 records remained for title and abstract screening. Of these, 81 were excluded (27 duplicate records and 54 unrelated studies), leaving 31 articles for full-text assessment. After full-text review, 23 articles were excluded for predefined reasons, and eight studies were finally included in the qualitative synthesis and meta-analysis. The screening process was conducted in accordance with the PRISMA guidelines (16). The eight remaining articles (17–24) were published between 2012 and 2022 and included 617 patients with sample sizes ranging from 11 to 252 patients. All articles were RCTs. Figure 1 shows the flow of the study selection process. Table 1 presents the characteristics of the studies included in this meta-analysis (Supplementary File 2).

**Table 1:** Characteristics of the included studies.

Trials	Study design	Type of patient	Duration (weeks)	Groups	Participants	Ages	Sex (male)	Comorbidities	Concomitant medications	Phosphate binders	Base 25(OH)D	Dose
Alshahawey et al. (17)	RCT	HD	12 weeks (3 months)	Treatment (cholecalciferol)	30	47 ± 10.65	50% (15/30)	Yes 1. Hypertension [9 (30%)] 2. Diabetes mellitus [2 (7%)] 3. Diabetes mellitus with complications [3 (10%)] 4. Ischemic cardiomyopathy [4 (13%)] 5. Tobacco use [3 (10%)] 6. Peripheral artery disease [1 (3%)]	Yes	Yes (not reported in detail)	17.9 (16.48–20) ng/ml	200.000 IU monthly for 3 months
				Control (placebo)	30	47.07 ± 12.35	53.33% (16/30)	Yes 1. Hypertension [7 (23%)] 2. Diabetes mellitus [3 (10%)] 3. Diabetes mellitus with complications [1 (3%)] 4. Ischemic cardiomyopathy [4 (13%)] 5. Tobacco use [2 (7%)] 6. Peripheral artery disease [1 (3%)]		Yes (not reported in detail)	18.7 (17.3–20.4) ng/ml	—
Ayub et al. (18)	RCT	HD	8 weeks (2 months)	Treatment (cholecalciferol)	35	49 ± 10.11	54.3% (19/35)	Yes 1. Diabetes [17 (48.6%)] 2. Hepatitis c [18 (51.4%)]	Yes	Yes (not reported in detail)	15.13 ± 5.34 ng/ml	25(OH)D: < 15 ng/ml → 50,000 IU weekly for 2 months; 16–30 ng/ml → 10,000 IU weekly for 2 months
				Control (placebo)	35	46 ± 14	65.7% (23/35)	Yes 1. Diabetes [17 (48.6%)] 2. Hepatitis c [17 (48.6%)]	Yes	Yes (not reported in detail)	17.03 ± 5.60 ng/ml	—
Gregório et al. (19)	RCT	HD	24 weeks (6 months)	Treatment (cholecalciferol)	18	59.0 (51.75,70.25)	50%	Yes 1. Diabetes (50%) 2. Hypertension(25%)	Yes	Yes 1. Calcium carbonate (33%) 2. Sevelamer(67%)	15.2 (10.6–23.1) ng/ml	Cholecalciferol 50.000 IU weekly for 3 month; if after 3 months 25(OH)D was ≥30 ng/ml, 50.000 IU was given monthly; If 25(OH)D was < 30 ng/ml, weekly administration was continued
				Control (placebo)	14	55.5 (50.50,65.25)	53%	Yes 1. Diabetes (46.7%) 2. Hypertensio(13.3%)	Yes	Yes 1. Calcium carbonate (29%) 2. Sevelamer(71%)	22.0 (17.2–24.1) ng/ml	—
Marckmann et al. (20)	RCT	HD	8 weeks	Treatment (cholecalciferol)	13	—	—	Unable to determine whether to use it or not	Yes	Unable to determine whether to use it or not	20.7 (16.3–28.9) nmol/L	40,000 IU weekly for 8 week
				Control (placebo)	14	—	—	Unable to determine whether to use it or not	Yes	Unable to determine whether to use it or not	35.9 (25.5–45.9) nmol/L	—
Miskulin et al. (21)	RCT	HD	24 weeks (6 months)	Treatment (ergocalciferol)	137	61.4 ± 13.3	—	Yes 1. Diabetes [66 (48%)] 2. Hypertension [39 (28%)]	Yes	Yes (not reported in detail, 85%)	16.0 ± 5.9 ng/ml	25(OH)D: ≤ 15 ng/ml → 50,000 IU weekly for 6 months; 16–30 ng/ml → 50,000 IU 50,000 IU weekly for 3 months, followed by 50,000 IU monthly for 3 months
				Control (placebo)	139	60.8 ± 13.9	—	Yes 1. Diabetes [61 (44%)] 2. Hypertensio [44 (32%)]	Yes	Yes (not reported in detail, 92%)	16.9 ± 6.4 ng/ml	—
Seibert et al. (22)	RCT	HD	12 weeks	Treatment (cholecalciferol)	15	66.9 ± 10.8	60.0% (9/15)	Yes (not reported in detail)	Yes	Yes 1. Ca-acetate [12 (4,635 mg/day)] 2. Algeldrate [4 (2,550 mg/day)] 3. Aluminum hydroxide [2 (900 mg/day)] 4. Sevelamer [4 (4,300mg/day)]	29.4 ± 11.2 nmol/L	25(OH)D: ≤ 12.5 nmol/L → 40,000 IU weekly for 12 weeks; 12.5–37.5 nmol/L → 20,000 IU weekly for 12 weeks; 37.5–75 nmol/L → 40,000 IU monthly for 12 weeks; 75–150 nmol/L → 20,000 IU monthly for 12 weeks
				Control (placebo)	18	67.4 ± 9.8	50.0% (9/18)	Yes (not reported in detail)	Yes	Yes 1. Ca-acetate [7 (3,686 mg/day)] 2. Algeldrate [4 (1,800 mg/day)] 3. Sevelamer [9 (3,689mg/day)]	33.6 ± 16.6 nmol/L	—
Sharif (23)	RCT	HD	8 weeks	Treatment (cholecalciferol)	11/31/6/5	59 ± 11.1	54.9% (28/51)	Yes 1. Diabetes [25 (49%)] 2. Hypertensio [23 (45%)] 3. Atherosclerotic disease [16 (31%)] 4. Acute coronary syndrome [12 (23%)] 5. CV congestion [5 (10%)] 6. Others (smoke) [23 (45%)]	Yes	No relevant description available	8.61 ± 0.8 ng/ml 12.66 ± 1.6 ng/ml 22.01 ± 2.36 ng/ml 31.25 ± 1.6 ng/ml	The primary dose of 0.25 mg/day for Cholecalciferol was determined on the basis of the levels of plasma calcium and parathyroid hormone (PTH). Dose adjustment for subsequent visits was determined accordingly up to a dose of 0.5 mg daily.
				Control (placebo)	10/30/6/5	56 ± 15.2	60.8% (31/53)	Yes 1. Diabetes [23 (45%)] 2. Hypertensio [27 (53%)] 3. Atherosclerotic disease [17 (33%)] 4. Acute coronary syndrome [13 (25%)] 5. CV congestion [7 (14%)] 6. Others (smoke) [28 (49%)]	Yes	No relevant description available	8.36 ± 0.35 ng/ml 12.70 ± 2.6 ng/ml 21.72 ± 1.3 ng/ml 30.46 ± 1.6 ng/ml	—
Tamadon et al. (24)	RCT	HD	12 weeks	Treatment (cholecalciferol)	30	60.1 ± 10.4	63.4% (19/30)	Yes Diabetes (100%)	Yes	Yes 1. Sevelamer [9 (30%)] 2. Calcium carbonate [21 (70%)]	15.2 ± 6.7 ng/ml	50,000 IU every 2 weeks for 12 weeks
				Control (placebo)	30	65.1 ± 10.1	63.4% (19/30)	Yes Diabetes (100%)	Yes	Yes 1. Sevelamer [8 (26.7%)] 2. Calcium carbonate [22 (73.3%)]	15.4 ± 6.9 ng/ml	—

Baseline 25(OH)D levels, dosing regimens, concomitant medications, and phosphate binders are presented as originally reported in the included studies to preserve the original study characteristics and reflect the clinical heterogeneity across trials. For the quantitative analyses, baseline 25(OH)D values were converted and harmonized to ng/ml, using the conversion factor of 1 ng/ml = 2.5 nmol/L. No re-categorization of dosing strategies or concomitant treatments was applied in this table.

### 3.2 Influence of vitamin D on the inflammation–nutrition factors

#### 3.2.1 CRP

**Figure 2:**
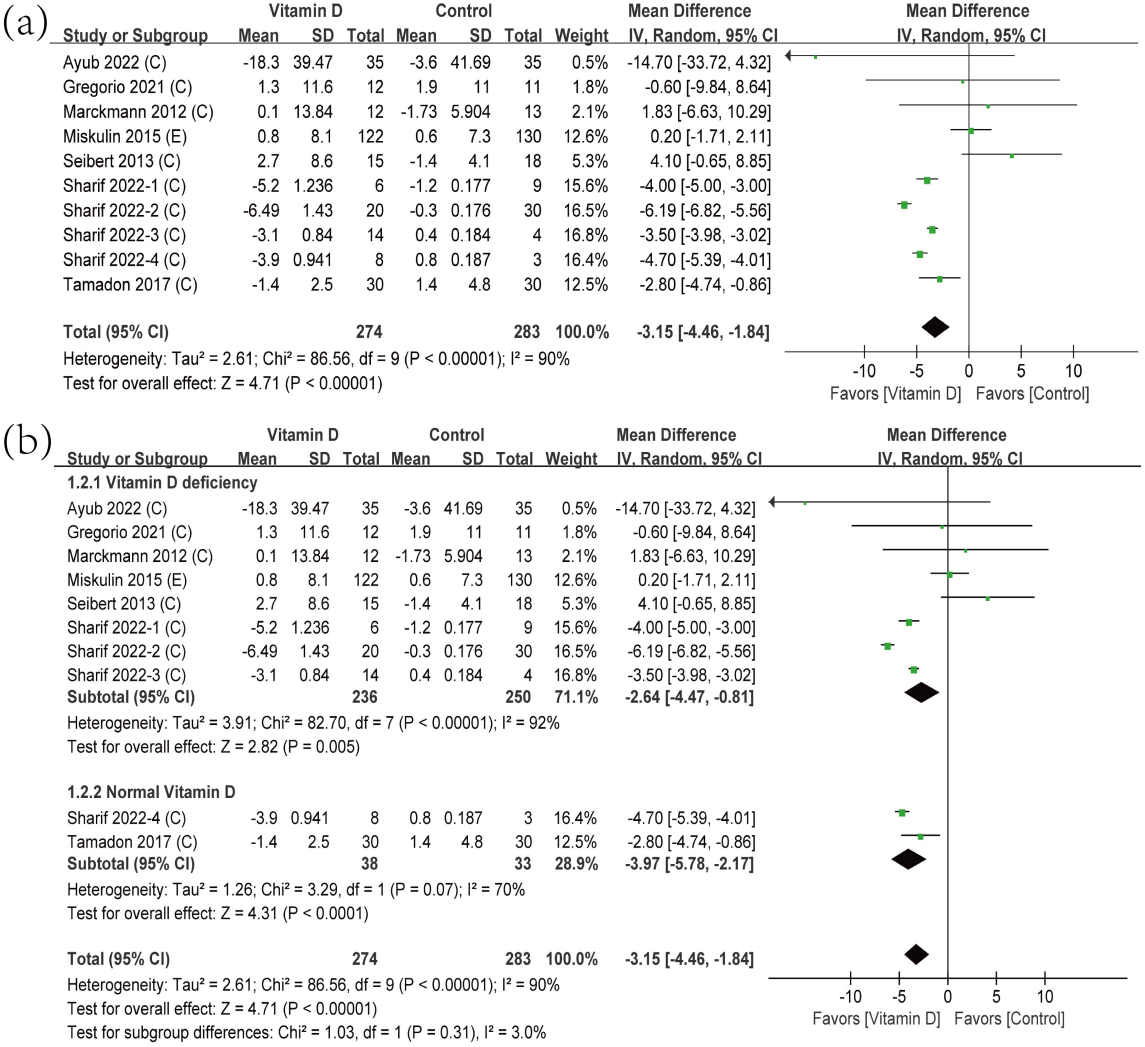
**(a)** Forest plot of the CRP change. **(b)** Forest plot of the CRP subgroup analysis by vitamin D deficiency status.

The seven randomized controlled trials (18–24) included reported CRP levels before and after treatment. Using the random-effects model, we found that compared with the control group, the decrease in CRP in the nutritional vitamin D intervention group was higher, yielding statistical significance [MD −3.15, 95% CI (−4.46, −1.84), *P* < 0.05] (Figure 2a).

We further evaluated the effect of nutritional vitamin D supplementation on CRP in the MHD population by conducting a subgroup analysis according to whether the baseline vitamin D was deficient. Nutritional vitamin D supplementation effectively reduced CRP both in patients with baseline vitamin D deficiency and in the MHD population with a normal vitamin D level, showing a statistical significance [MD −2.64, 95% CI (−4.47, −0.81), *P* < 0.05; MD −3.97, 95% CI (−5.78, −2.17), *P* < 0.05] (Figure 2b).

The other non-classical clinical indicators related to inflammation were also compared and analyzed. Compared with the control group, nutritional vitamin D supplementation could reduce the pro-inflammatory factors such as TNF-α, IL-1β, white blood cell count (WBC), but did not get statistical significance (*P* = 0.08, *P* = 0.44, *P* = 0.32; Supplementary materials 2–4 in Supplementary File 1).

#### 3.2.2 Albumin

**Figure 3:**
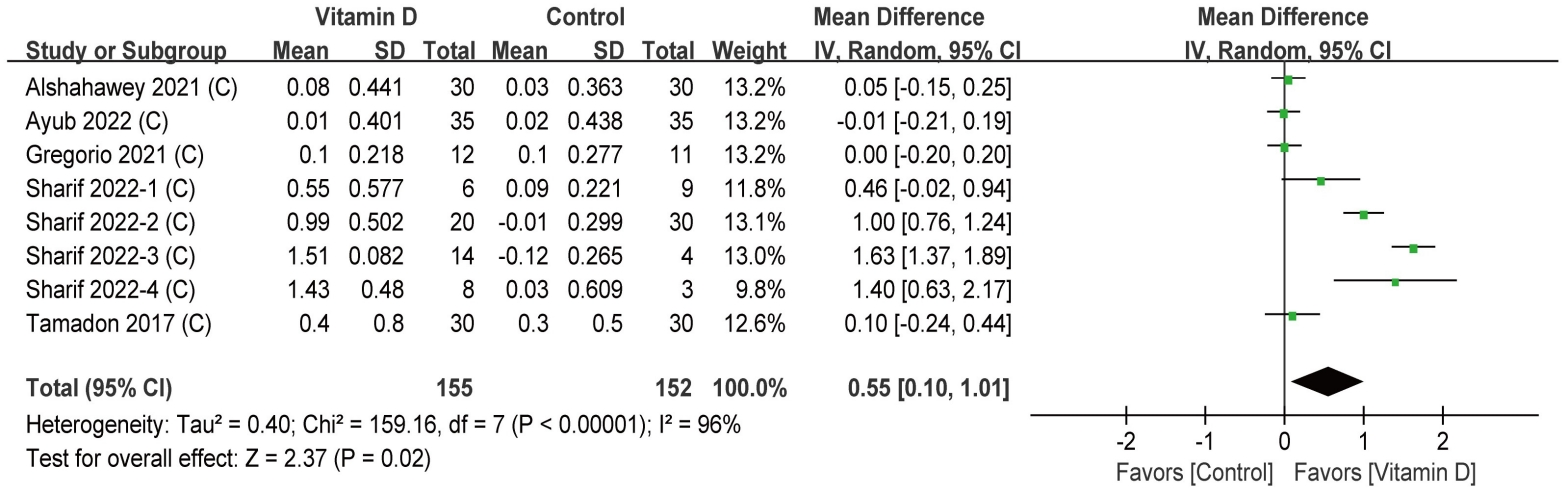
Forest plot of the albumin change.

Five RCT studies (17–19, 23, 24) described the changes in serum albumin before and after the vitamin D supplementation. The changes in serum albumin were analyzed by using the random-effects model. The nutritional vitamin D intervention more significantly im-proved the serum albumin level of the MHD patients compared to the control group, showing statistical significance [MD 0.55, 95% CI (0.10, 1.01), *P* = 0.02] (Figure 3).

### 3.3 Effects on vitamin D

**Figure 4:**
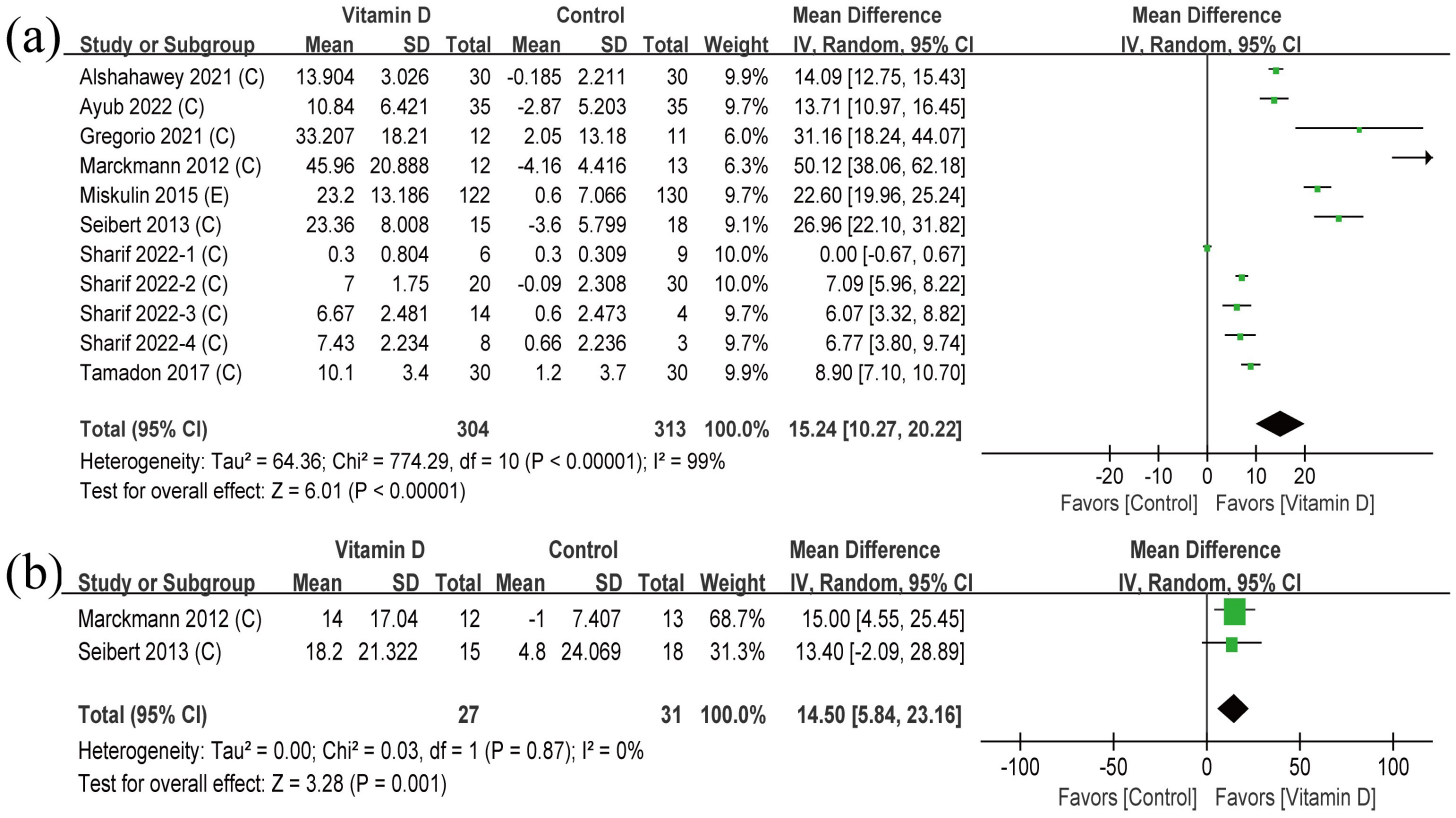
**(a)** Forest plot showing the change in 25(OH)D. **(b)** Forest plot showing the change in 1,25(OH)_2_D.

The changes in 25(OH)D before and after the nutritional vitamin D intervention were recorded in eight studies (17–24), and two of them (20, 22) reported changes in 1,25(OH)_2_D levels. Using random–effects models, we found that after the nutritional vitamin D supplementation, the elevations in 25(OH)D and 1,25(OH)_2_D levels in the vitamin D intervention group were significantly higher than those in the control group [MD 15.24, 95% CI (10.27, 20.22), *P* < 0.05], [MD 14.50, 95% CI (5.84, 23.16), *P* = 0.001] (Figures 4a, b).

### 3.4 Changes in the chronic kidney disease–mineral bone disease (CKD–MBD) related indicators

#### 3.4.1 Phosphate

**Figure 5:**
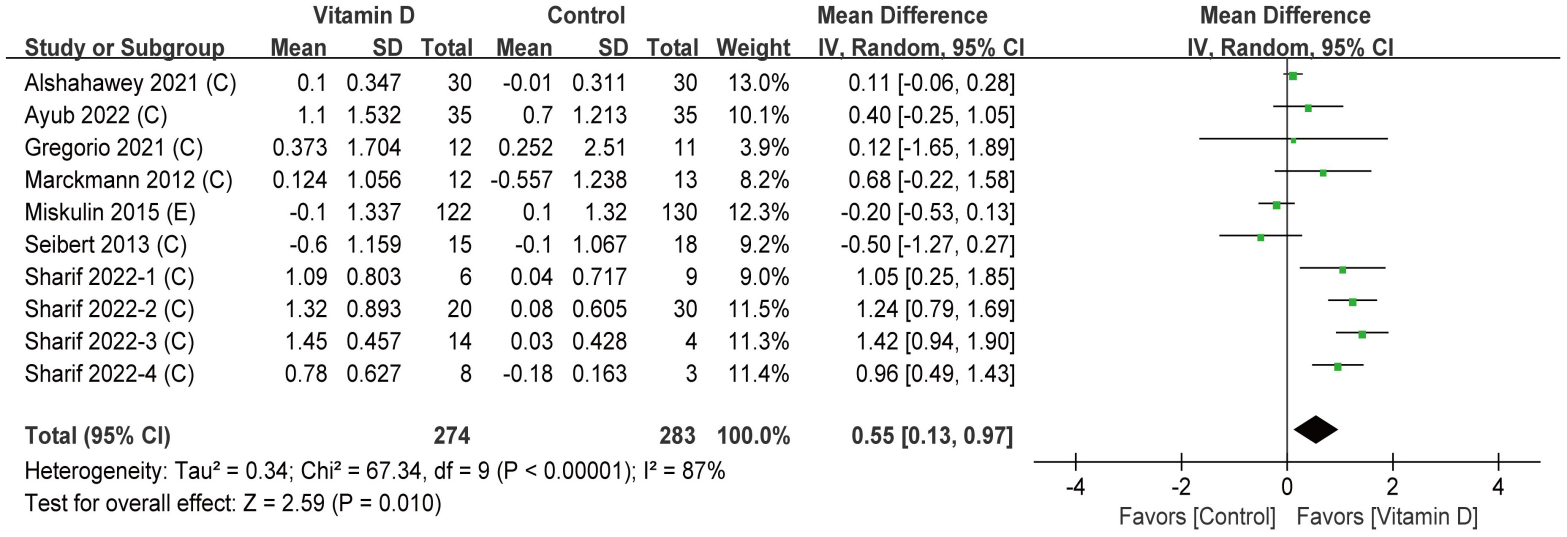
Forest plot of phosphate.

Except for Tamadon 2017, the other seven included papers (17–23) recorded the changes in phosphate before and after treatment. The random-effect model was used for the analysis. The increase in blood phosphorus in the nutritional vitamin D intervention group was significantly higher than that in the control group [MD 0.55, 95% CI (0.13, 0.97), *P* = 0.01] (Figure 5).

#### 3.4.2 Calcium

**Figure 6:**
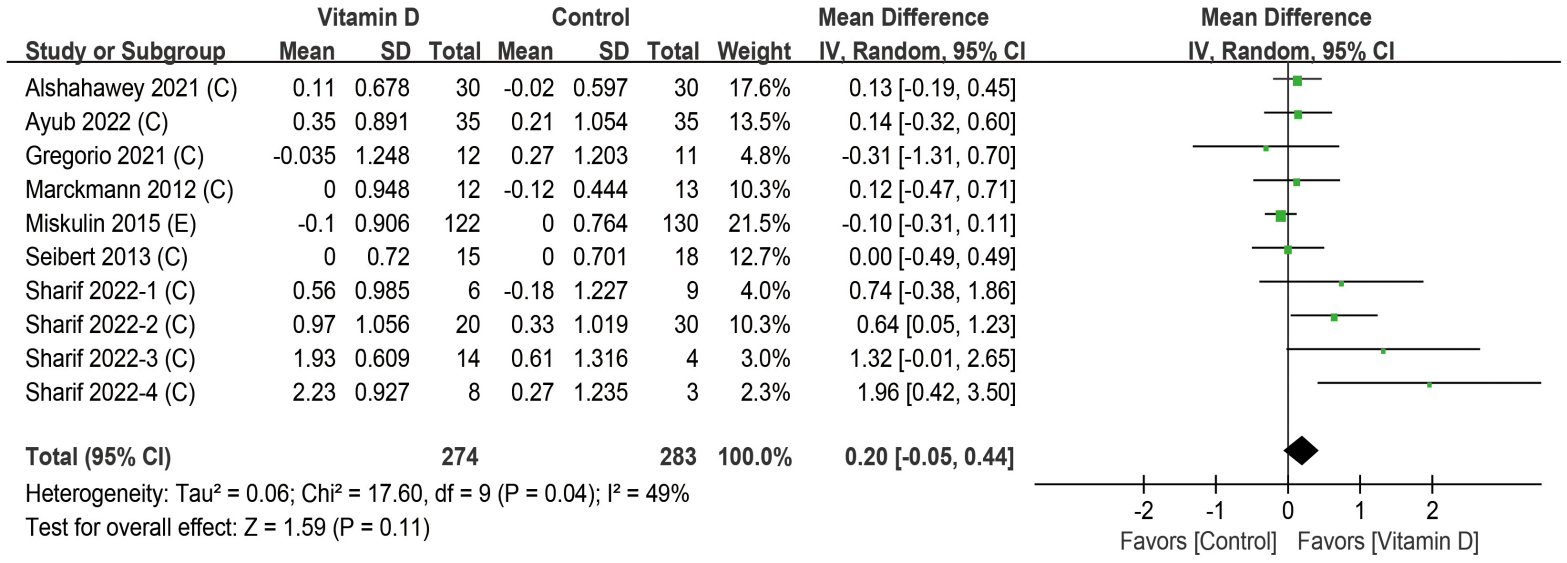
Forest plot for calcium.

The random-effect model was used to analyze the effect of the nutritional vitamin D intervention on calcium (17–23). The results showed that the increase in blood calcium in the intervention group was similar to that in the control group, depicting no statistical significance (*P* = 0.11; Figure 6).

#### 3.4.3 iPTH

**Figure 7:**
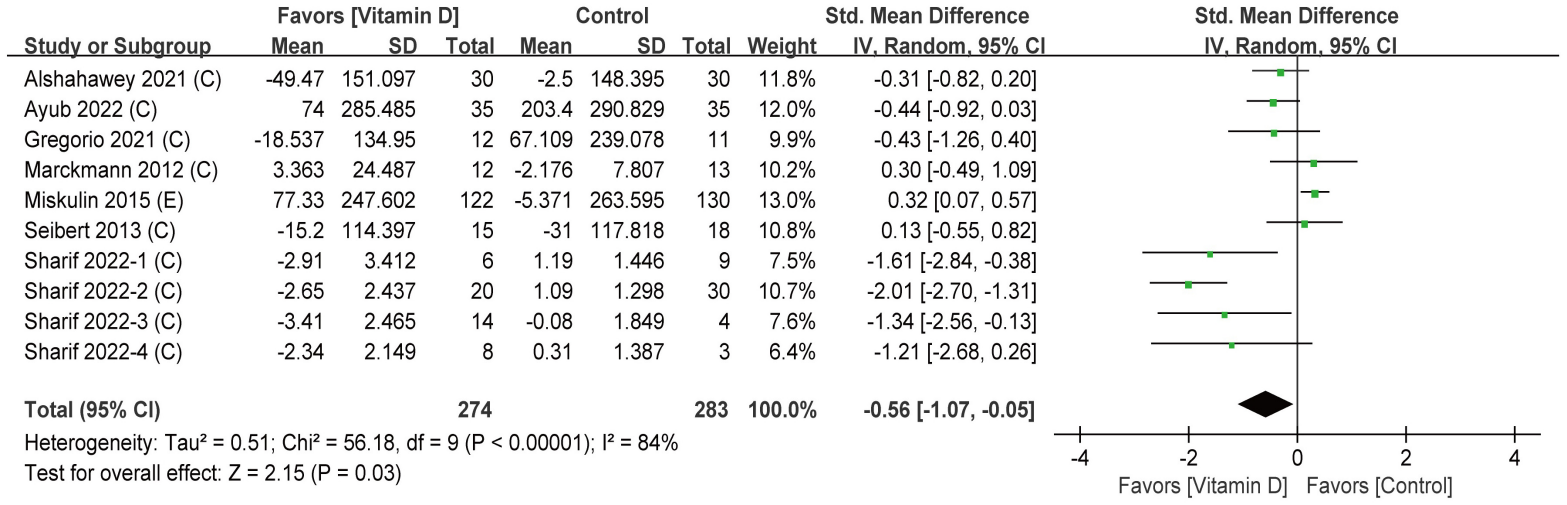
Forest plot showing the change in iPTH levels.

Seven RCT studies (17–23) recorded the changes in iPTH levels before and after nutrition-al vitamin D treatment. The random-effects model showed that, compared with the control group, the intervention with nutritional vitamin D significantly reduced parathyroid hormone levels in MHD patients [SMD −0.56, 95% CI (−1.07, −0.05), *P* = 0.03] (Figure 7).

#### 3.4.4 Alkaline phosphatase (ALP)

**Figure 8:**
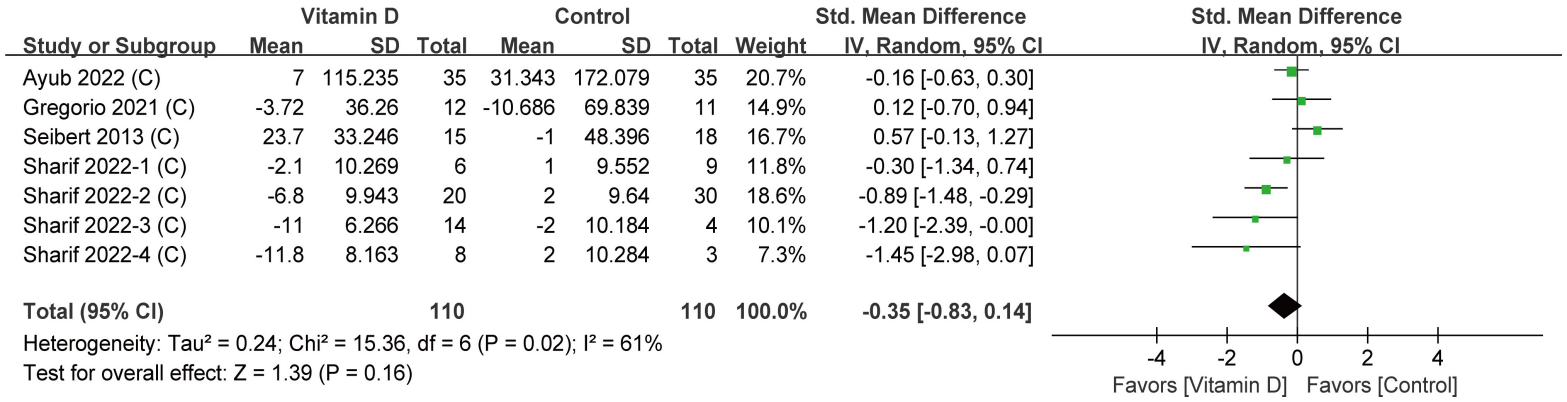
Forest plot showing the change in ALP levels.

Four RCT studies (18, 19, 22, 23) recorded the changes in the ALP levels before and after the vitamin D intervention. Using the random-effects model, we found no significant difference in the reduction of ALP between the intervention and control groups [SMD −0.35, 95% CI (−0.83, 0.14), *P* = 0.16] (Figure 8).

#### 3.4.5 FGF-23

**Figure 9:**
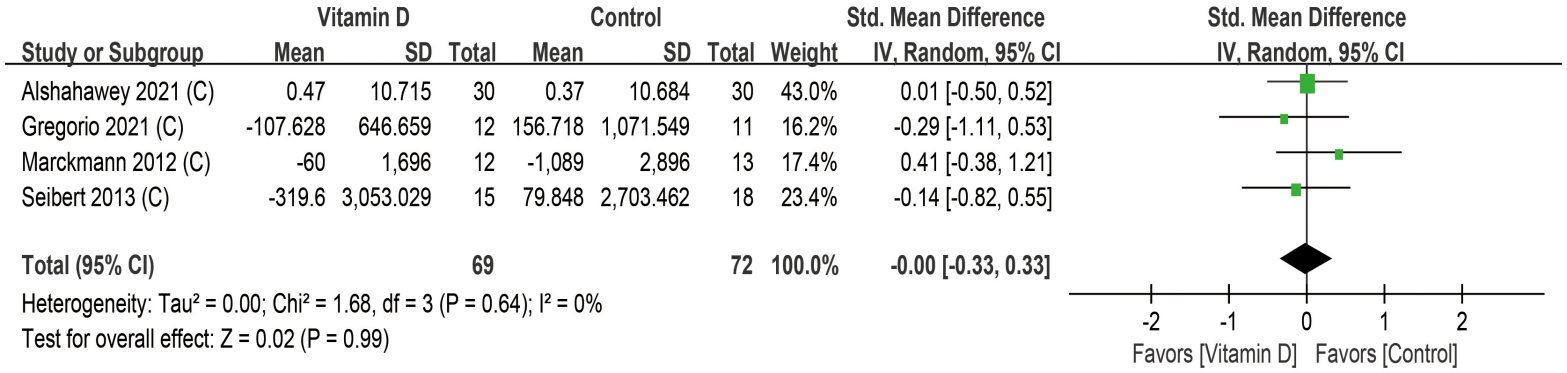
Forest plot showing the change in FGF-23.

Four RCTs (17, 18, 20, 22) showed changes in FGF-23 levels. Compared with the control group, the decrease in FGF-23 in the vitamin D intervention group was greater; however, no significant difference was found between the two groups [SMD −0.00, 95% CI (−0.33, 0.33), *P* = 0.99] (Figure 9).

### 3.5 Safety analysis

**Figure 10:**
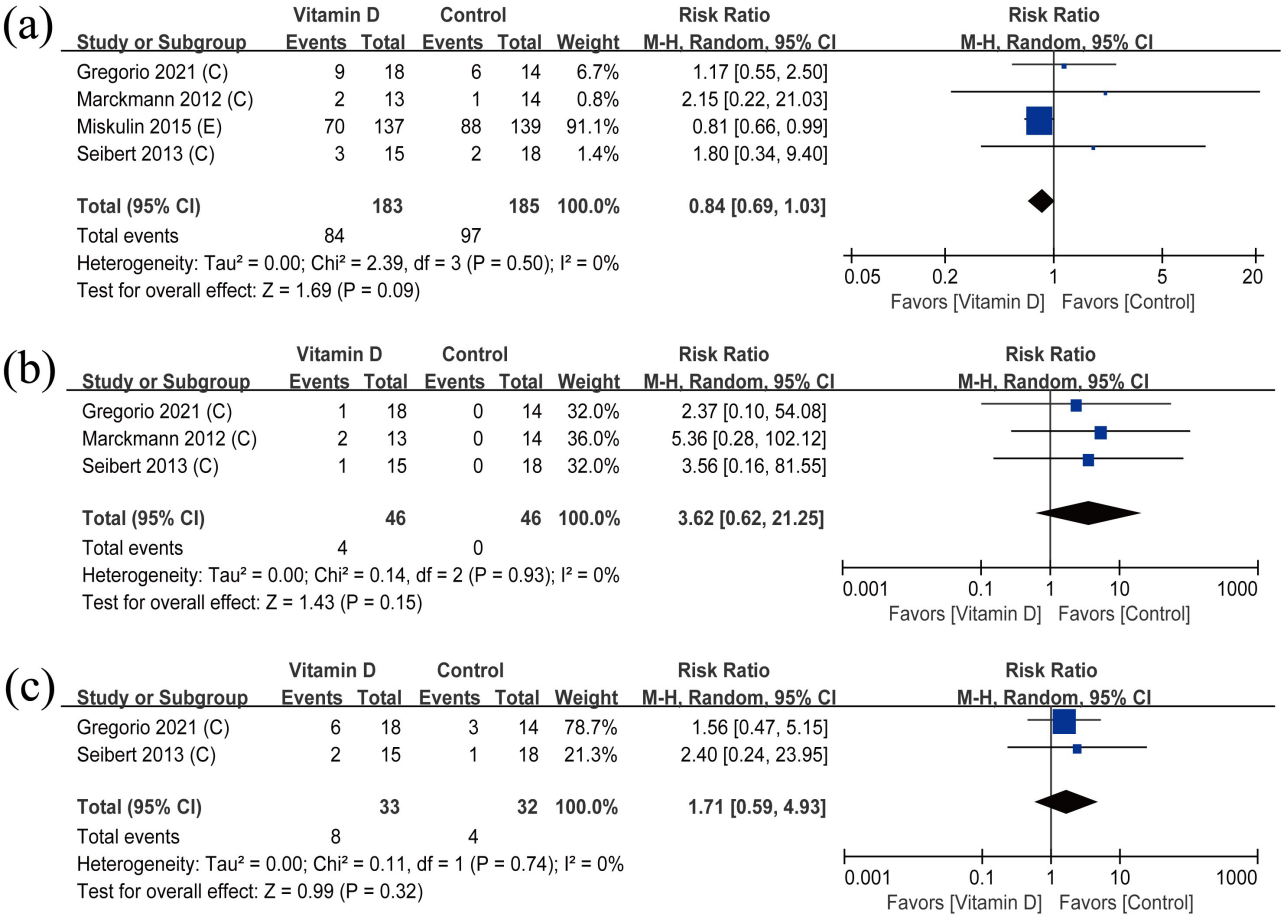
**(a)** Forest plot for TEAEs. **(b)** Forest plot for hypercalcemia. **(c)** Forest plot for hyperphosphatemia.

Four RCTs (19–22) recorded the number of adverse events in the vitamin D and placebo groups, including vomiting, falling, infection-related hospitalization, hyperphosphatemia, and hypercalcemia. The random-effects model results showed that the TEAEs in the nutrition-al vitamin D intervention group were higher than those in the control group; however, no significant difference was derived (RR 0.84, 95% CI [0.69, 1.03], *P* = 0.09; Figure 10a). The incidence of hypercalcemia (19, 20, 22) and hyperphosphatemia (19, 22) in the treatment group was higher than that in the control group, but no statistical significance was found (*P* = 0.15, *P* = 0.32; Figures 10b, c).

### 3.6 Risk analysis of bias in the included studies

**Figure 11:**
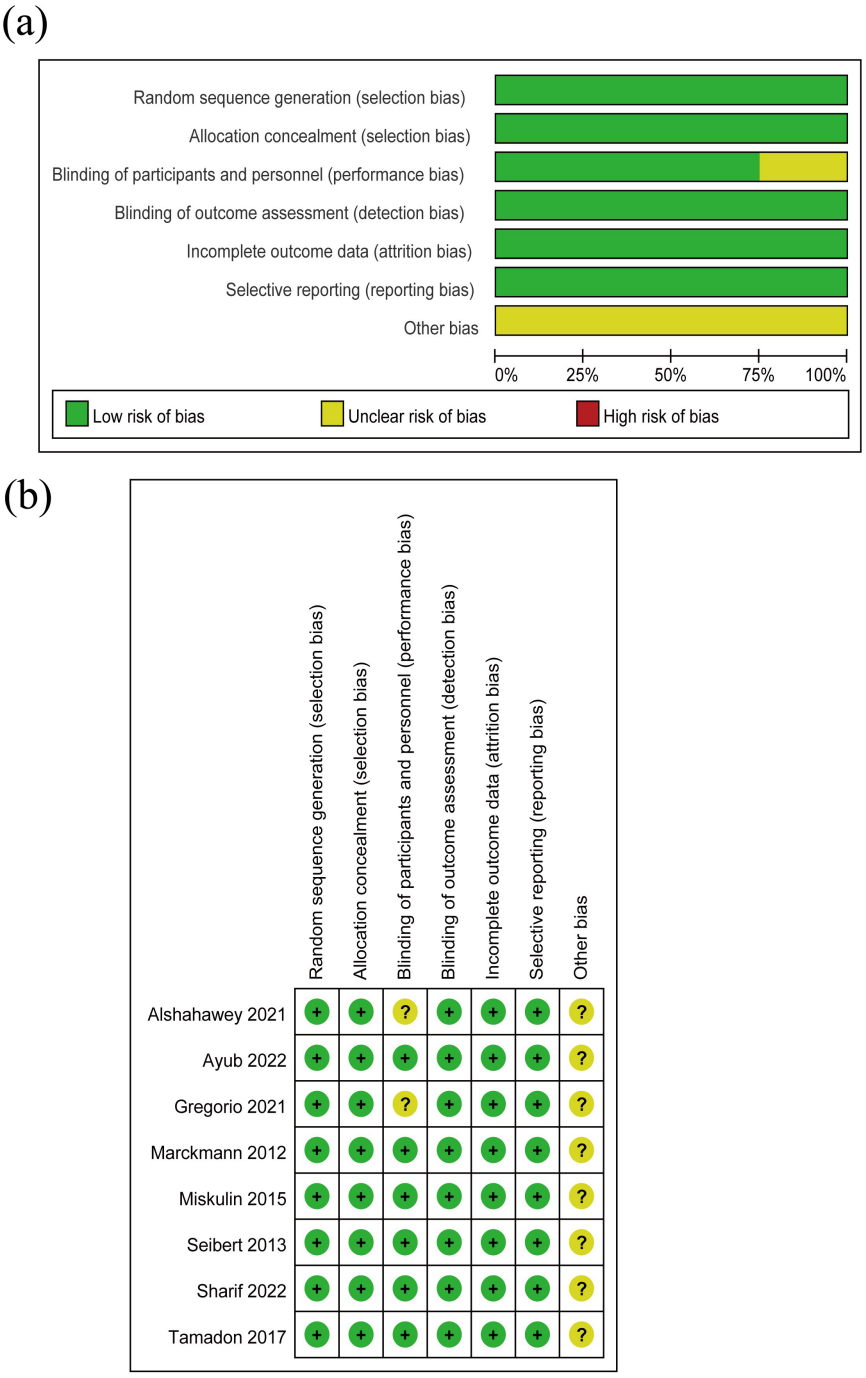
**(a)** Risk of bias graph. **(b)** Risk of bias summary.

Risk of bias was assessed using the Cochrane risk of bias framework in RevMan 5.4.1. The included RCTs were evaluated across the domains of random sequence generation, allocation concealment, blinding of participants and personnel, blinding of outcome assessment, completeness of outcome data, selective reporting, and other bias. Each domain was judged as low, unclear, or high risk of bias. As shown in Figure 11, most studies were assessed as low risk in several domains, whereas Gregório et al. (19) and Alshahawey et al. (17) were judged as unclear risk in the domain of blinding of participants and personnel. In addition, all included studies were rated as unclear risk for other bias.

### 3.7 Publication bias assessment of the included studies

**Figure 12:**
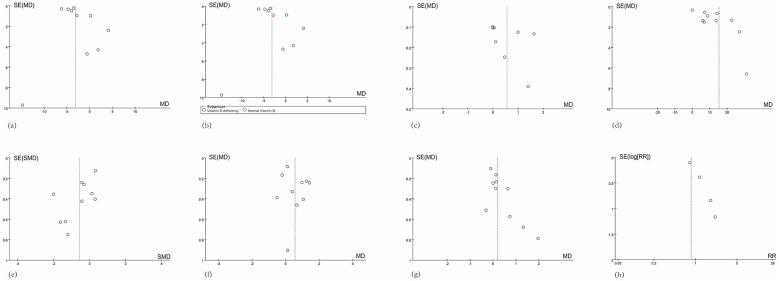
Funnel plot on publication bias. **(a)** CRP. **(b)** CRP by vitamin D deficiency status. **(c)** Albumin. **(d)** 25(OH)D. **(e)** iPTH. **(f)** phosphate. **(g)** calcium. **(h)** TEAEs.

**Figure 13:**
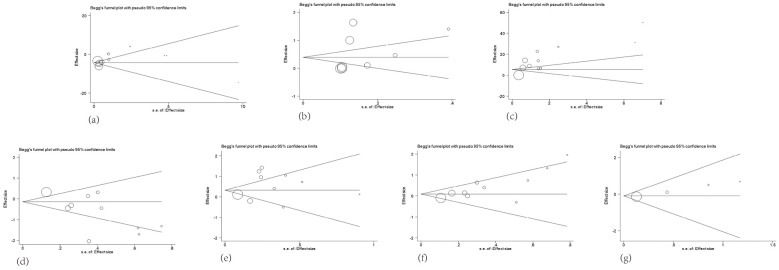
Begg's funnel plot. **(a)** CRP. **(b)** Albumin. **(c)** 25(OH)D. **(d)** iPTH. **(e)** phosphate. **(f)** calcium. **(g)** TEAEs.

The funnel chart generated using RevMan 5.4.1 software was used to evaluate the publication bias of the included studies. Figure 12 depicts the funnel diagram. We further per-formed Begg's test on key indicators of visual asymmetry using Stata 17 software. The results showed *P* values of 1 (CRP), 0.108 (albumin), 0.533 (25(OH)D), 0.152 (iPTH), 0.858 (Phosphate), and 0.049 (Calcium), 0.308 (TEAEs; Figure 13). No obvious evidence of publication bias was observed for the other outcomes; however, these findings should be interpreted with caution given the limited number of included studies. Potential publication bias was suggested for calcium. Nevertheless, the pooled results for calcium were not materially altered after trim-and-fill analysis, with the direction of effect remaining unchanged and the magnitude of the combined effect showing no substantial difference, suggesting that the result was relatively stable (Supplementary material 5 in Supplementary File 1). In addition, the certainty of evidence for each outcome was assessed using the Grading of Recommendations Assessment, Development and Evaluation (GRADE) approach, with the corresponding ratings provided in Supplementary material 7 in Supplementary Files 1 and 4.

### 3.8 Sensitivity analysis

We used Stata 17 to conduct sensitivity analysis on key outcome indicators with a high heterogeneity to evaluate the robustness of the results. In conclusion, the analysis results of the CRP and 25(OH)D were both robust. The heterogeneity of the combined results of albumin was due to the difference in the sample sizes between Sharif (2022-2) and Sharif (2022-4) in the subgroups of the Sharif (23) study. Sensitivity analysis excluding studies in which medians and interquartile ranges were converted to means and standard deviations further confirmed the robustness of the results (Supplementary material 6 in Supplementary File 1). In addition, Hartung–Knapp–Sidik–Jonkman (HKSJ)-adjusted analyses were performed as supplementary sensitivity analyses, and the results are presented in Supplementary File 5.

## 4 Discussion

A persistent microinflammation is a hidden pathological state observed in the MHD patients. It was firstly proposed by Schömig et al. (25). The incidence of a microinflammatory status in the MHD patients can reach 30%−50% (26), which is an important pathophysiological mechanism causing and exacerbating malnutrition. Microinflammation and malnutrition can have a cause-and-effect relationship with each other, consequently forming a vicious circle and causing atherosclerosis through various mechanisms. Malnutrition–inflammation–atherosclerosis syndrome is the main cause of the high incidence of cardio-vascular events and the all-cause death in MHD patients (27). Therefore, the effective prevention and treatment of microinflammation–malnutrition have an important clinical significance for improving the long-term prognosis of the MHD population.

A large-scale study found that up to 78% of newly diagnosed dialysis patients have vitamin D deficiency (28). Low vitamin D is often manifested as low 25(OH)D levels. When dialysis patients are in a state of sustained low 25(OH)D levels, oxidative stress and inflammation in the body are added. Liu et al. (29) pointed out that vitamin D deficiency may promote immune dysregulation and inflammatory responses in CKD/ESRD, while supplementation with nutritional vitamin D in hemodialysis patients can still increase 25(OH)D and 1,25(OH)2D levels, suggesting that vitamin D may remain biologically active even during dialysis through extra-renal activation and immune modulation, thereby providing mechanistic support for its potential role in improving microinflammation in MHD patients. However, it remains unclear whether or not the exogenous supplementation of nutritional vitamin D can improve the microinflammatory status of the MHD patients. CRP is one of the most commonly used biomarkers for assessing inflammatory status in this population and is also widely used in clinical practice as an indicator of microinflammation (30, 31). In this meta-analysis, nutritional vitamin D supplementation was associated with a reduction in CRP levels,3.15mg/L, in patients undergoing MHD, combining with the down trend in TNF-α and IL-1β levels. Based on this finding, we further performed subgroup analyses and found that the CRP-lowering effect of exogenous nutritional vitamin D supplementation was observed not only in MHD patients with vitamin D deficiency, but also in those with relatively normal baseline vitamin D levels, suggesting that the effect is not limited to patients with overt vitamin D deficiency. Subgroup analyses, according to vitamin D type and treatment duration, showed that nutritional vitamin D supplementation exhibited a more pronounced effect on reducing CRP during short-term treatment, whereas the differences among different types of vitamin D were small (Supplementary materials 1.1, 1.2 in Supplementary File 1). Regarding cumulative dose, among the only two studies with calculable cumulative doses, broadly similar total cumulative vitamin D doses were associated with inconsistent CRP outcomes, with one study showing no significant reduction and the other showing a significant decrease. Therefore, further large-scale, well-designed RCTs are needed to clarify the potential impact of cumulative vitamin D dose on CRP response (Supplementary material 1.3 in Supplementary File 1). However, previous studies have shown that a weekly dosing regimen can significantly reduce CRP and neutrophil-to-lymphocyte ratio (NLR) levels and improve oxidative stress markers in MHD patients, whereas the benefits of monthly pulse dosing appear to be relatively limited (32). Nevertheless, because the included studies mainly reported laboratory inflammatory markers rather than more direct clinical outcomes, and the clinical significance based on the observed CRP reduction and the actual benefits require further confirmation in more high-quality clinical studies. In addition, serum albumin levels were significantly increased in the vitamin D administration group. Although serum albumin is an important predictor of malnutrition in MHD patients, it is also influenced by various non-nutritional factors, including volume status, infection, and inflammation. Therefore, the increase in albumin suggests that nutritional vitamin D supplementation has the potential to improve the nutrition–inflammation status, of MHD patients, and further large-scale studies are still needed to clarify this issue.

CKD–MBD is one of the important complications suffered by the MHD population. In addition to bone disease, it is manifested as a mineral metabolism disorder, which is an important pathogenic mechanism for increasing the cardiovascular disease risk in MHD patients. Vitamin D and its metabolites play important roles in mineral and bone metabolism in patients on maintenance hemodialysis. Previous studies have shown that nutritional vitamin D deficiency is common in the MHD population (33), and similar findings have also been reported in conference abstract literature (34). However, whether or not the deficiency of vitamin D corrected by exogenous supplementation can play a therapeutic role in CKD–MBD in the MHD population remains unclear. This meta-analysis conducted that the nutritional vitamin D administration significantly increased the 25(OH)D level in the MHD population and the active vitamin D level at the same time. The iPTH, ALP, and FGF-23 levels, the direct parameters of CKD-MBD, showed a downward trend in the MHD population treated with the exogenous nutritional vitamin D, with no significant increase in blood calcium level. The trends of these indicators suggest that the bone metabolism of the MHD patients is improved after the nutritional vitamin D administration. However, whether or not the nutritional vitamin D supplementation has a synergistic effect on control of the key indicators of CKD-MBD in the MHD patients remains to be further clarified. It is worth mentioning that this study found higher blood phosphorus levels in patients receiving nutritional vitamin D intervention compared to those without intervention. The reason is considered to be related to increased gastrointestinal phosphorus absorption due to elevated active vitamin D levels after nutritional vitamin D supplementation. It cannot be ruled out that differences in factors directly affecting blood phosphorus levels, such as dietary control, phosphate binder dosage, monitoring frequency, and adjustment frequency between the two patient groups, also contributed. Since these relevant details were not recorded in detail for all included studies. Even though the safety assessment showed no increase in hyperphosphatemia incidence despite elevated blood phosphorus levels, the potential for drug-related phosphorus elevation in the clinical application of nutritional vitamin D for MHD patients still requires thorough consideration. Adjustments to monitoring frequency, phosphate-lowering medications, and active vitamin D dosage in prescriptions may be necessary when needed.

In terms of safety, the nutritional vitamin D treatment did not increase the overall incidence of adverse events. Vitamin D intervention did not significantly improve the blood calcium level or the incidence of hypercalcemia. The blood phosphorus level of the MHD patients with the nutritional vitamin D supplementation increased more significantly, but the incidence of hyperphosphatemia was not higher than that of the placebo, suggesting that the exogenous administration of nutritional vitamin D in the MHD population is generally safe.

There are several limitations in our meta-analysis that should be considered. First, because only a limited number of studies were included in this meta-analysis and the sample size of each study was relatively small, the statistical power, robustness, and generalizability of our findings may be limited. Second, not all RCTs have observed enough indicators related to inflammation (e.g., TNF-α and IL-1β); hence, the data included in the analysis of the inflammation–malnutrition indicators, which are of interest to clinicians, are not sufficiently rich. Third, not all studies included detailed records and descriptions of the conventional treatment drugs used by the MHD patients during the RCT process (e.g., active vitamin D and its analogs, phosphate-lowering drugs, and calcium-mimetic agents). To some extent, these weakened the conclusion drawn from our study as regards the intervention effect of the nutritional vitamin D supplementation on CKD–MBD. Finally, the treatment duration of the trials fluctuated from 8 to 24 weeks and was not sufficient for evaluating the therapeutic effect and associated complications more precisely. Therefore, the increased duration of the drug administration and the subsequent follow-up must be considered in future clinical studies.

## 5 Conclusions

In summary, the present meta-analysis supports a beneficial association between nutritional vitamin D supplementation and improvements in inflammation–nutrition-related markers in the MHD population. The findings suggest that nutritional vitamin D may exert a favorable anti-inflammatory effect in patients undergoing maintenance hemodialysis, without evidence of an increased incidence of hypercalcemia or hyperphosphatemia in the included studies. However, the certainty of the current evidence remains limited, and the effects of nutritional vitamin D on bone metabolism and more direct clinical outcomes require further confirmation in large-scale, multicenter, and rigorously designed randomized controlled trials.

## Data Availability

The data presented in the study are included in the article/Supplementary material. The final, consolidated dataset generated and analyzed during the current study (including the extracted and converted values) has been deposited in the Figshare repository and is publicly available via the following link: https://figshare.com/articles/dataset/My_first_meta_data/30259174.
