# Supplementary material for: The role of nutritional vitamin D on microinflammation and nutritional status in maintenance hemodialysis patients: a meta-analysis of randomized controlled trials
Source: Front Nutr. 2026 May 29;13:1767616. doi: 10.3389/fnut.2026.1767616 (PMC13260404; doi:10.3389/fnut.2026.1767616)
Supplement: Supplementary file 1 [file Supplementary_file_1.docx]

Supplementary File 1

# Additional analyses by vitamin D type, cumulative dose, and treatment duration

## Type of vitamin D

After summarizing the vitamin D interventions in the experimental groups from the included RCTs (see Table 1), we confirmed that the interventions were limited to Cholecalciferol (vitamin D3; the same compound) and Ergocalciferol (vitamin D2). Among the seven included RCTs, only one trial (Miskulin 2015) used Ergocalciferol. Therefore, we conducted a sensitivity analysis. After excluding this D2 trial, the results are shown in the figure below. The combined effect on CRP remained significant and consistent with the original direction, while the effect size slightly increased (MD changed from -3.15 to -3.75), indicating robust conclusions. However, further clinical studies involving Ergocalciferol are needed to eliminate potential heterogeneity in CRP reduction caused by vitamin D types.
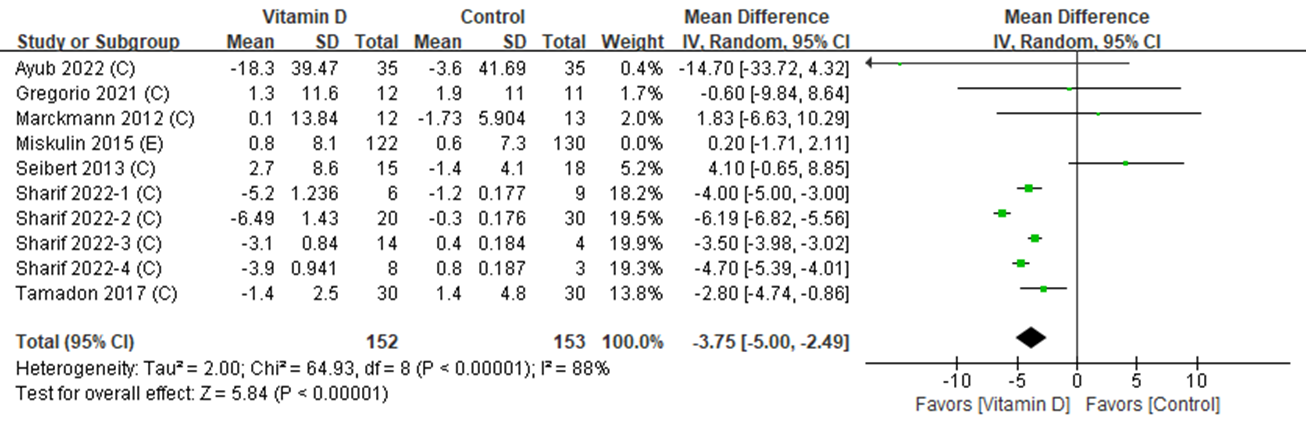


**Figure S1.** Forest plot for change in CRP after excluding Miskulin 2015.

## Subgroup analysis according to treatment duration

We conducted a subgroup analysis on CRP based on treatment duration (≤ 12 weeks,>12 weeks), and the results indicated that subgroup difference testing suggested that treatment duration may be a potential influencing factor (p=0.0006<0.001). When the intervention period was ≤ 12 weeks, the experimental group showed a significant decrease in CRP compared to the control group, which was statistically significant (p<0.001). However, when the intervention period was>12 weeks, although the results suggested that the experimental group could reduce CRP levels, they were not significant or statistically significant. Perhaps a shorter course of treatment may indicate that the effect of vitamin D on inflammatory indicators mainly occurs in the early stages of intervention, with a plateau effect on the decrease of CRP with the supplementation of vitamin D. At the same time, the limited number of studies may also become a potential influencing factor for insignificant research results, so more clinical studies with different treatment durations are still needed to support the decreasing effect of vitamin D on CRP.


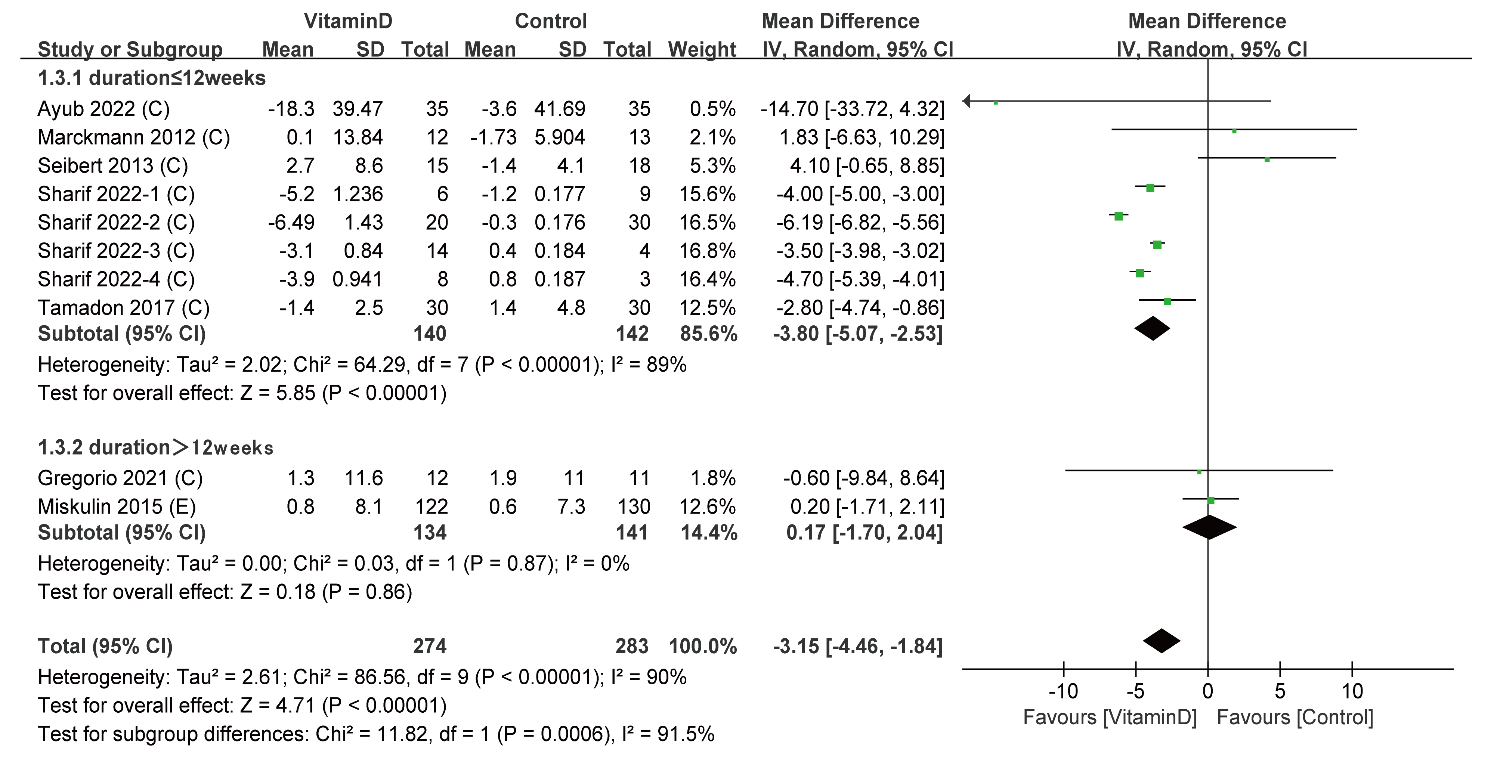


**Figure S2.** Forest plot for change in CRP by treatment duration.

## Exploratory analysis of cumulative vitamin D dose and changes in CRP

Cumulative vitamin D dose was assessed as a potential source of heterogeneity in CRP response across the included studies. Considerable variation was observed in dosing strategies. Two trials (Gregorio, 2021; Sharif, 2022) used dose-adjustment protocols based on serum calcium and parathyroid hormone levels, whereas three trials (Ayub, 2022; Miskulin, 2015; Seibert, 2013) adopted stratified supplementation according to baseline vitamin D status. Because these studies did not report sufficient information on the proportion of participants in each dose category or the average dose actually achieved, cumulative dose could not be reliably estimated for most trials.Among the included studies, only two trials, Marckmann (2012) and Tamadon (2017), used fixed-dose regimens that allowed calculation of total cumulative dose. Marckmann (2012) administered a cumulative dose of 320,000 IU over 8 weeks and showed no significant reduction in CRP [MD 1.83, 95% CI (-6.63, 10.29)], whereas Tamadon (2017) administered 300,000 IU over 12 weeks and demonstrated a significant reduction in CRP [MD -2.80, 95% CI (-4.74, -0.86)].Despite broadly similar cumulative doses, the two studies yielded different CRP outcomes, suggesting that cumulative dose alone may not fully account for the observed variation in CRP response. Other factors, including treatment duration, dosing schedule, vitamin D formulation, and clinical characteristics of the study populations, may also have contributed. Further well-designed clinical studies with clearly reported cumulative dosing data are needed to clarify the potential impact of cumulative vitamin D dose on CRP response in patients undergoing maintenance.

| **Study** | **Duration** | **Cumulative dose (IU)** | **CRP change** |
| --- | --- | --- | --- |
| Marckmann, 2012 | 8 weeks | 320,000 | MD 1.83 (95% CI -6.63 to 10.29) |
| Tamadon, 2017 | 12 weeks | 300,000 | MD -2.80 (95% CI -4.74 to -0.86) |

**Table S1.** Cumulative dose summary.

# TNF-α

Two RCTs [22,23] introduced the changes of TNF - α levels before and after treatment. TNF - α is also one of the indicators of micro inflammatory state, which is often elevated in dialysis patients. The random effect model was used to analyze it. Obviously, compared with the control group, the experimental group could reduce the serum level of TNF - α and reduce the inflammatory state, but it was not statistically significant (MD -0.65,95% CI [-1.38,0.07], P = 0.08).

**
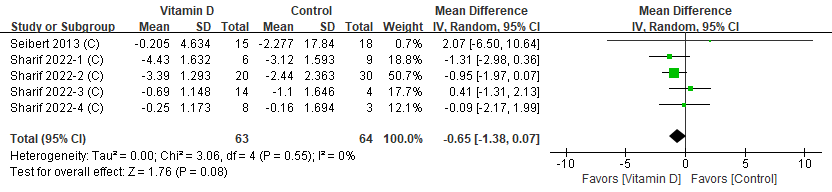
**

**Figure S3.** Forest plot for change in TNF-α

# IL-1β

Two studies [19,23] recorded the difference changes of IL-1 β before and after the test. Compared with the control group, nutritional vitamin D supplementation could reduce the pro-inflammatory factor IL-1 β, but there was no statistical significance. (SMD -0.42,95% CI [-1.50,0.65], P = 0.44).


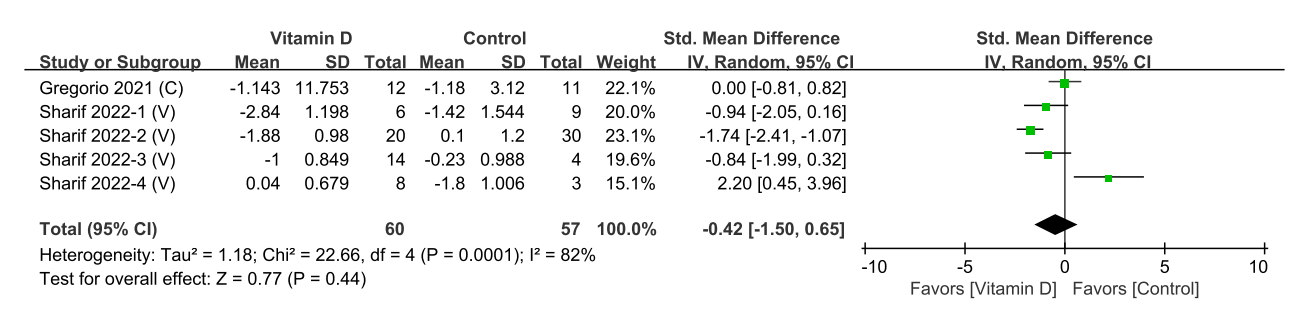


**Figure S4.** Forest plot for change in IL-1β

# WBC

Two studies [18,19] recorded the difference changes of WBC before and after the test. Compared with the control group, nutritional vitamin D supplementation could reduce WBC, but there was no statistical significance (MD -0.43,95% CI [-1.30,0.43], P = 0.32).


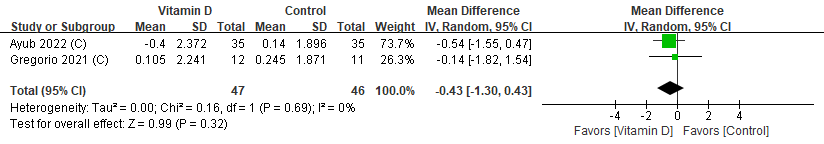


**Figure S5.** Forest plot for change in WBC

# Comparison of results before and after adjusting the indicator "calcium" using the shear compensation method

| 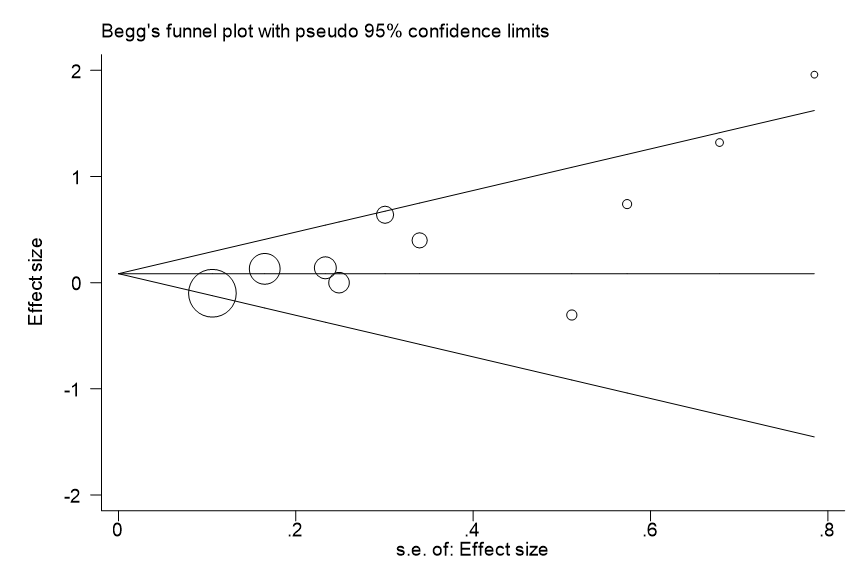 | 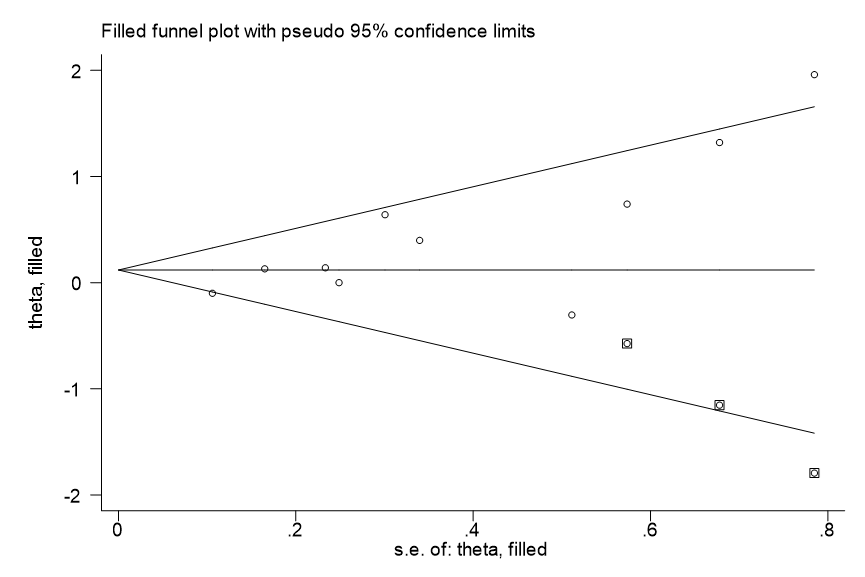 |
| --- | --- |
| (a) | (b) |

**Figure S6.** Comparison of results before and after adjusting the indicator "calcium" using the shear compensation method (a)Results before using the pruning method. (b) The result after using the pruning method.

# Sensitivity analysis


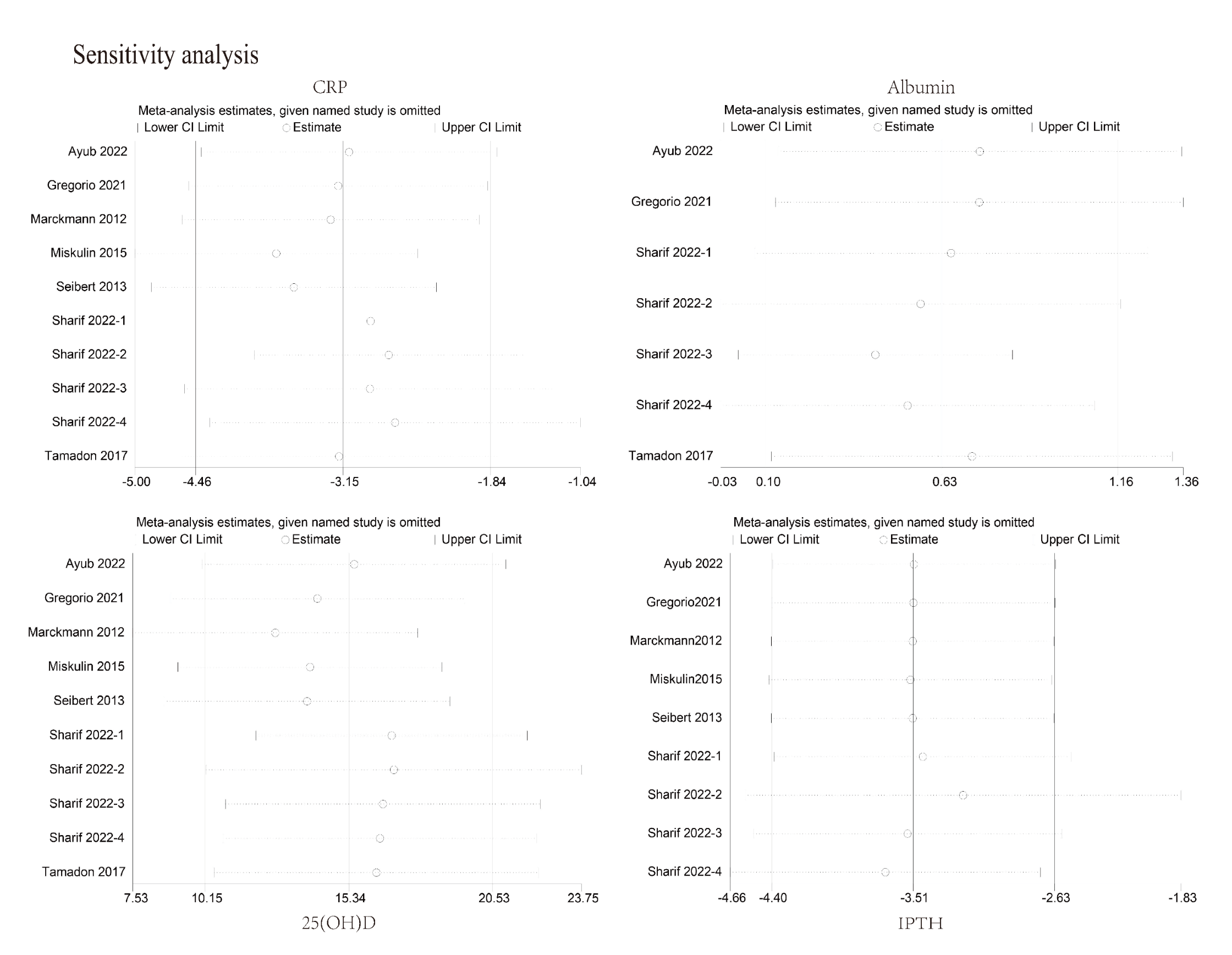


**Figure S7.** leave-one-out sensitivity analysis

| CRP  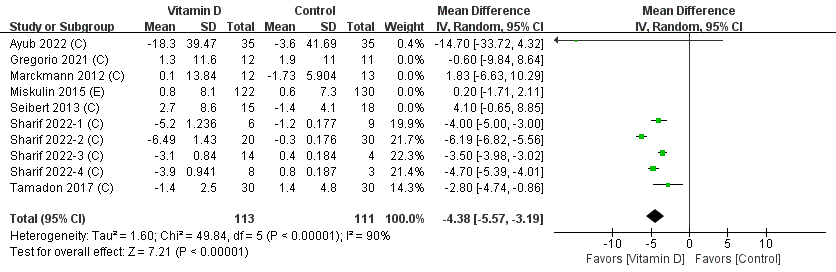 | 25(OH)D  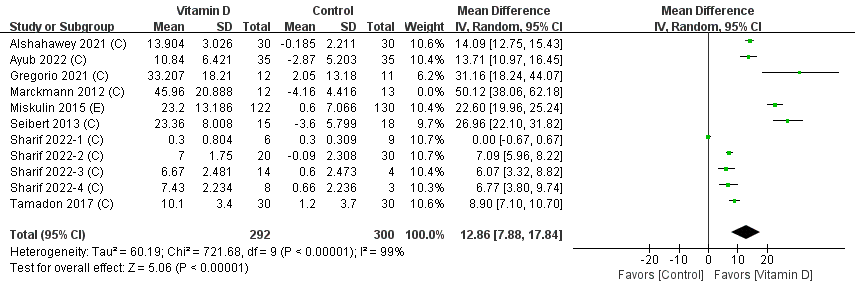 |
| --- | --- |
| Calcium  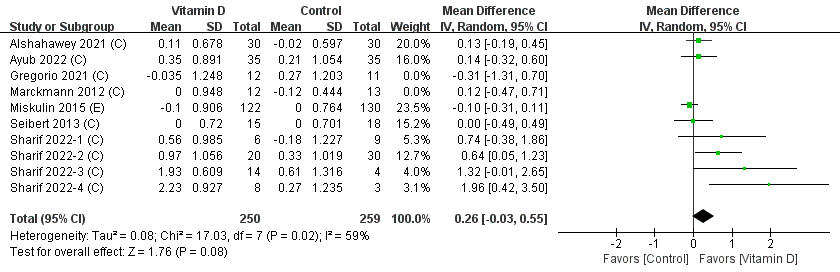 | Phosphate  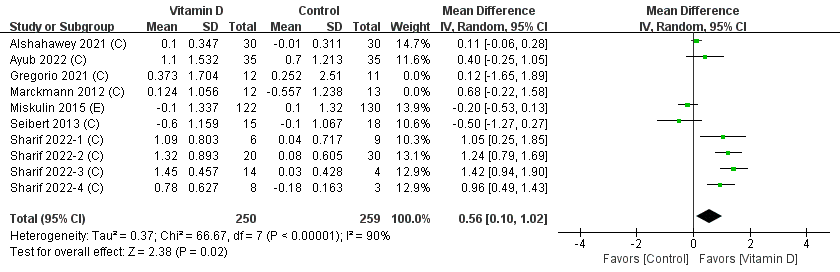 |
| IPTH  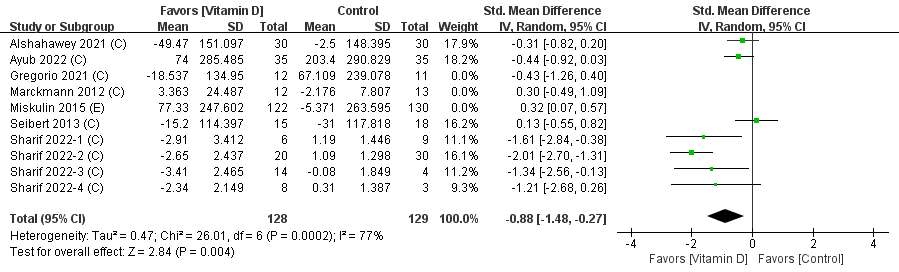 | ALP  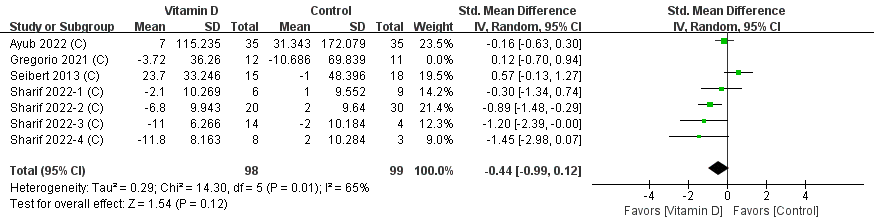 |
| TNF-α  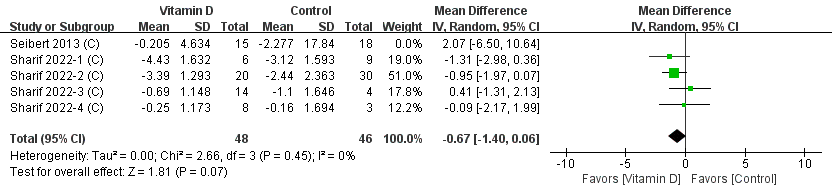 | IL-1β  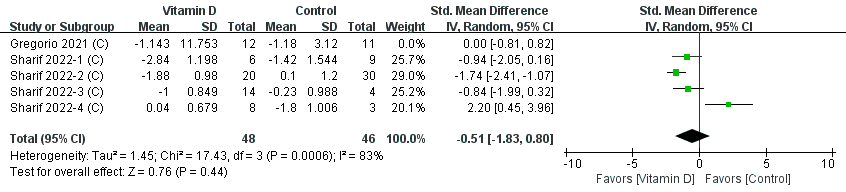 |

**Figure S8.** Sensitivity analysis excluding studies in which medians and interquartile ranges were converted to means and standard deviations. No significant changes were observed in the results of various indicators.

# GRADE

| 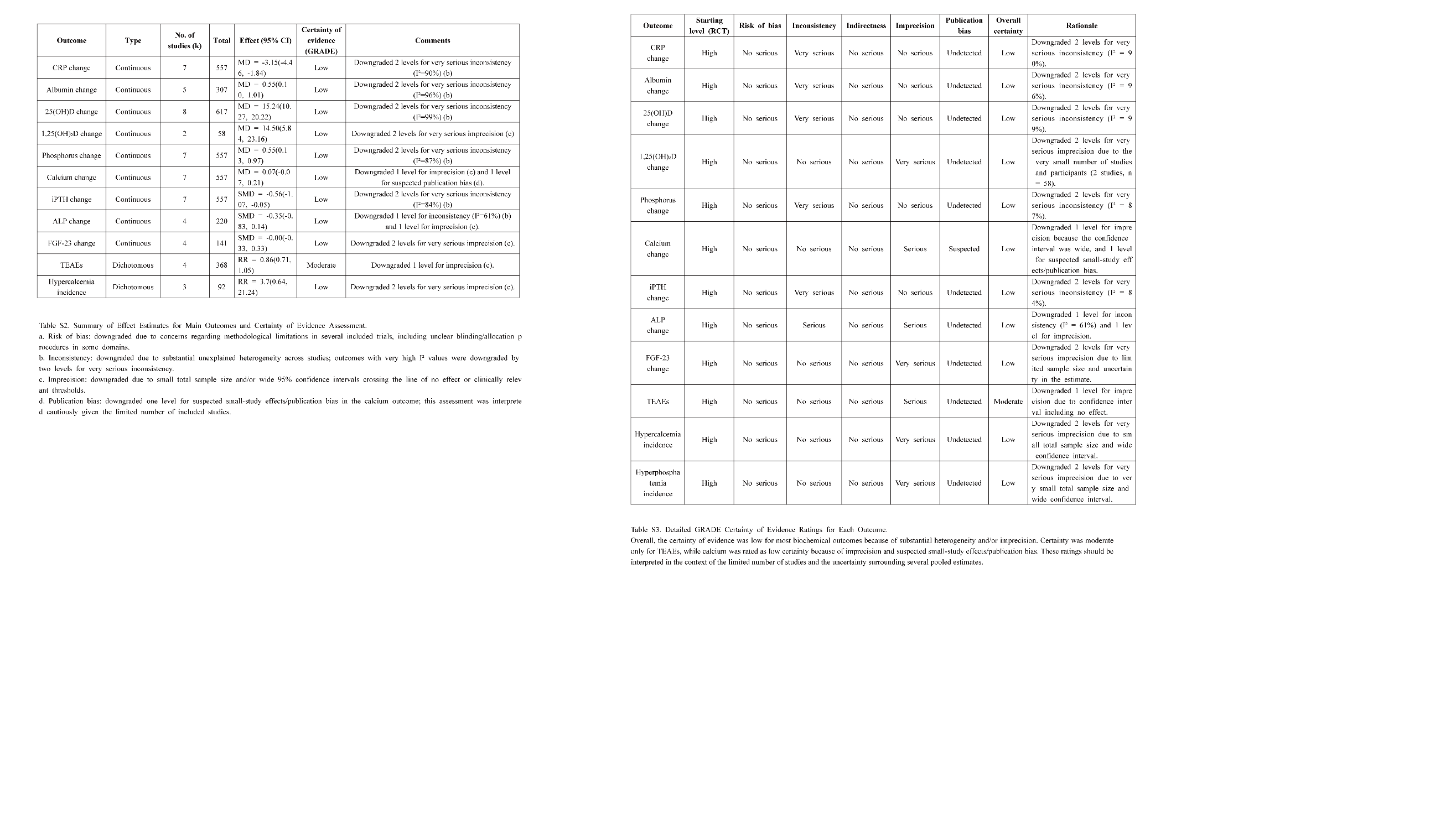 |
| --- |
| **Table S2.** Summary of Effect Estimates for Main Outcomes and Certainty of Evidence Assessment.  a. Risk of bias: downgraded due to concerns regarding methodological limitations in several included trials, including unclear blinding/allocation procedures in some domains. b. Inconsistency: downgraded due to substantial unexplained heterogeneity across studies; outcomes with very high I² values were downgraded by two levels for very serious inconsistency. c. Imprecision: downgraded due to small total sample size and/or wide 95% confidence intervals crossing the line of no effect or clinically relevant thresholds. d. Publication bias: downgraded one level for suspected small-study effects/publication bias in the calcium outcome; this assessment was interpreted cautiously given the limited number of included studies. |
| 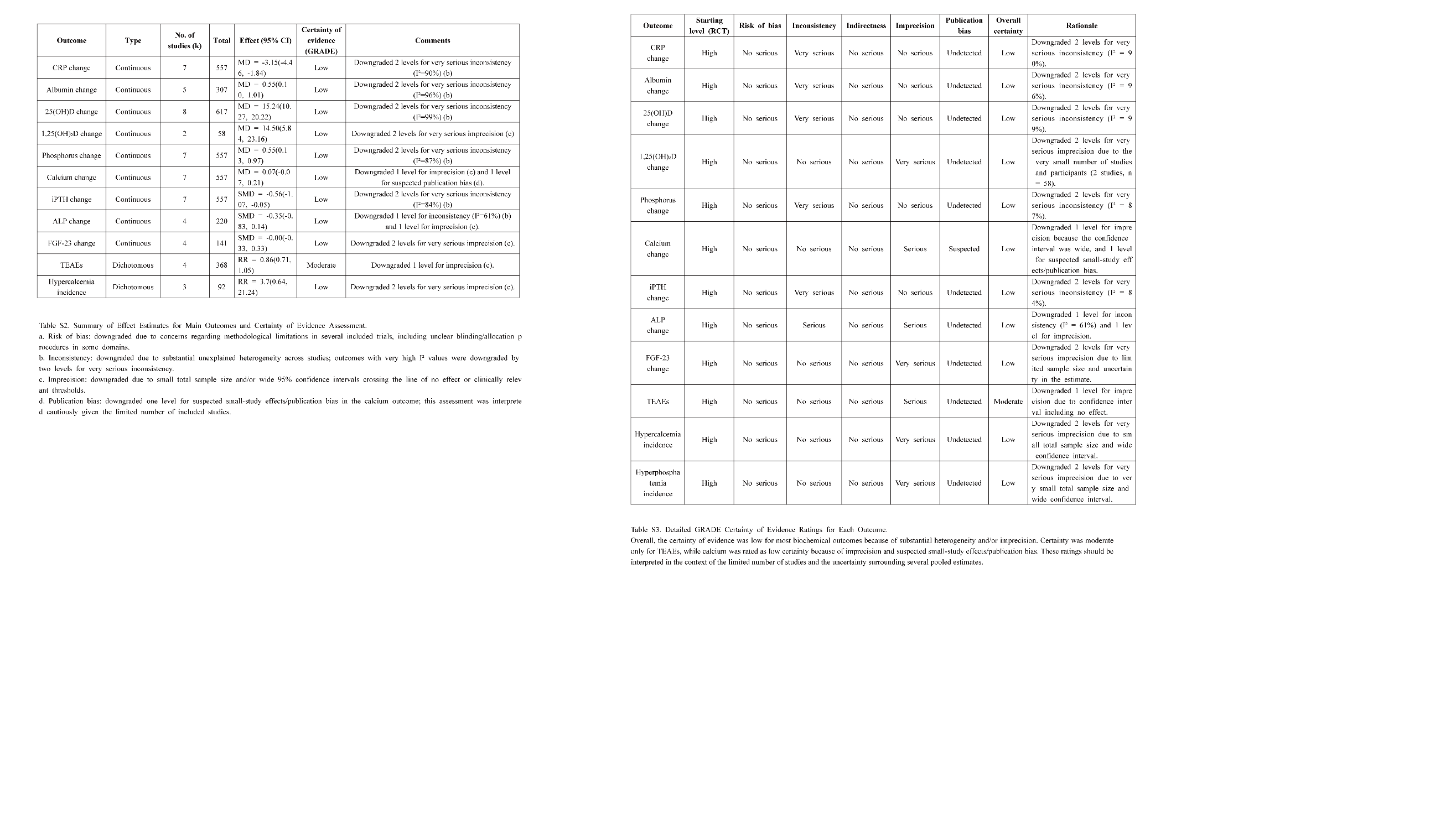 |
| **Table S3.** Detailed GRADE Certainty of Evidence Ratings for Each Outcome.  Overall, the certainty of evidence was low for most biochemical outcomes because of substantial heterogeneity and/or imprecision. Certainty was moderate only for TEAEs, while calcium was rated as low certainty because of imprecision and suspected small-study effects/publication bias. These ratings should be interpreted in the context of the limited number of studies and the uncertainty surrounding several pooled estimates. |

# 8. Search strategy

PubMed

1. Vitamin D-related terms

("Vitamin D"[Mesh] OR "Receptors, Calcitriol"[Mesh] OR "Ergocalciferols"[Mesh] OR "Cholecalciferol"[Mesh] OR Calciferols OR "Vitamin D 2" OR Ergocalciferol OR Vitamin D2 OR "D2, Vitamin" OR "1,25-Dihydroxycholecalciferol Receptor" OR "1,25 Dihydroxycholecalciferol Receptor" OR "Receptor, 1,25-Dihydroxycholecalciferol" OR "1,25-Dihydroxycholecalciferol Receptors" OR "1,25 Dihydroxycholecalciferol Receptors" OR "Receptors, 1,25-Dihydroxycholecalciferol" OR "Vitamin D3 Receptor" OR "D3 Receptor, Vitamin" OR "Receptor, Vitamin D3" OR "Vitamin D3 Receptors" OR "Receptors, Vitamin D3" OR "D3 Receptors, Vitamin" OR "1,25-Dihydroxyvitamin D3 Receptor" OR "1,25 Dihydroxyvitamin D3 Receptor" OR "D3 Receptor, 1,25-Dihydroxyvitamin" OR "Receptor, 1,25-Dihydroxyvitamin D3" OR "1,25-Dihydroxyvitamin D3 Receptors" OR "1,25 Dihydroxyvitamin D3 Receptors" OR "D3 Receptors, 1,25-Dihydroxyvitamin" OR "Receptors, 1,25-Dihydroxyvitamin D3" OR "Calcitriol Receptor" OR "Receptor, Calcitriol" OR "Calcitriol Receptors" OR "Receptors, Cholecalciferol" OR "Cholecalciferol Receptors" OR "Vitamin D Receptor" OR "D Receptor, Vitamin" OR "Receptor, Vitamin D" OR "Receptors, Vitamin D" OR "D Receptors, Vitamin" OR "Vitamin D Receptors" OR "1,25-Dihydroxyvitamin D 3 Receptor" OR "1,25 Dihydroxyvitamin D 3 Receptor" OR "Receptors, 1,25-Dihydroxyvitamin D 3" OR "Receptors, Vitamin D 3" OR "Vitamin D 3 Receptor" OR "Vitamin D 3 Receptors" OR "Vitamin D 3" OR Vitamin D3 OR "(3 beta,5Z,7E)-9,10-Secocholesta-5,7,10(19)-trien-3-ol" OR Calciol OR Cholecalciferols)

**Records retrieved:** 83,882

1. Inflammation-related terms

("Inflammation"[Mesh] OR Inflammations OR "Innate Inflammatory Response" OR "Inflammatory Response, Innate" OR "Innate Inflammatory Responses" OR "Inflammation Mediators"[Mesh] OR "Mediators, Inflammation" OR "Mediators of Inflammation")

**Records retrieved:** 1,367,882

1. Chronic kidney disease / dialysis-related terms

("Renal Insufficiency, Chronic"[Mesh] OR "Chronic Renal Insufficiencies" OR "Renal Insufficiencies, Chronic" OR "Chronic Kidney Insufficiency" OR "Chronic Kidney Insufficiencies" OR "Kidney Insufficiencies, Chronic" OR "Chronic Renal Insufficiency" OR "Kidney Insufficiency, Chronic" OR "Chronic Kidney Diseases" OR "Chronic Kidney Disease" OR "Disease, Chronic Kidney" OR "Diseases, Chronic Kidney" OR "Kidney Disease, Chronic" OR "Kidney Diseases, Chronic" OR "Chronic Renal Diseases" OR "Chronic Renal Disease" OR "Disease, Chronic Renal" OR "Diseases, Chronic Renal" OR "Renal Disease, Chronic" OR "Renal Diseases, Chronic" OR "Renal Dialysis"[Mesh] OR "Dialyses, Renal" OR "Renal Dialyses" OR "Dialysis, Renal" OR Hemodialysis OR Hemodialyses OR "Dialysis, Extracorporeal" OR "Dialyses, Extracorporeal" OR "Extracorporeal Dialyses" OR "Extracorporeal Dialysis")

**Records retrieved**: 350,262

1) and 2) and 3) =332

Web of Science

1. Vitamin D-related terms

TS=("Vitamin D" OR Calcitriol OR Ergocalciferols OR Calciferols OR "Vitamin D 2" OR Ergocalciferol OR Vitamin D2 OR "1,25-Dihydroxycholecalciferol Receptor" OR "1,25 Dihydroxycholecalciferol Receptor" OR "1,25-Dihydroxycholecalciferol Receptors" OR "1,25 Dihydroxycholecalciferol Receptors" OR "Vitamin D3 Receptor" OR "Vitamin D3 Receptors" OR "1,25-Dihydroxyvitamin D3 Receptor" OR "1,25 Dihydroxyvitamin D3 Receptor" OR "1,25-Dihydroxyvitamin D3 Receptors" OR "1,25 Dihydroxyvitamin D3 Receptors" OR "Calcitriol Receptor" OR "Calcitriol Receptors" OR "Cholecalciferol Receptors" OR "Vitamin D Receptor" OR "Vitamin D Receptors" OR "1,25-Dihydroxyvitamin D 3 Receptor" OR "1,25 Dihydroxyvitamin D 3 Receptor" OR "Vitamin D 3 Receptor" OR "Vitamin D 3 Receptors" OR Cholecalciferol OR "Vitamin D 3" OR Vitamin D3)

**Records retrieved:** 222,215

1. Inflammation-related terms

TS=(inflammat* OR "Innate Inflammatory Response" OR "Innate Inflammatory Responses" OR "Inflammation Mediators" OR "Mediators of Inflammation")

**Records retrieved:** 2,626,746

1. Chronic kidney disease / dialysis-related terms

TS=("Chronic Renal Insufficiencies" OR "Chronic Kidney Insufficiency" OR "Chronic Kidney Insufficiencies" OR "Chronic Renal Insufficiency" OR "Chronic Kidney Diseases" OR "Chronic Kidney Disease" OR "Chronic Renal Diseases" OR "Chronic Renal Disease" OR "Renal Dialysis" OR "Renal Dialyses" OR "Dialysis, Renal" OR Hemodialysis OR Hemodialyses OR "Extracorporeal Dialyses" OR "Extracorporeal Dialysis")

**Records retrieved:** 326,184

4) and 5) and 6) =1,302
