# Supplementary material for: The role of nutritional vitamin D on microinflammation and nutritional status in maintenance hemodialysis patients: a meta-analysis of randomized controlled trials
Source: Front Nutr. 2026 May 29;13:1767616. doi: 10.3389/fnut.2026.1767616 (PMC13260404; doi:10.3389/fnut.2026.1767616)
Supplement: Supplementary file 2 [file Supplementary_file_2.docx]

Supplementary File 2

Table 1. Characteristics of the included studies

| **Trials** | **Study Design** | **Type of patient** | **Duration**  **(weeks)** | **Groups** | **Participants** | **Ages** | **Sex**  **(Male)** | **comorbidities** | **concomitant**  **medications** | **phosphate**  **binders** | **Base**  **25(OH)D** | **Dose** |
| --- | --- | --- | --- | --- | --- | --- | --- | --- | --- | --- | --- | --- |
| Alshahawey  et al.,2021[17] | RCT | HD | 12w(3m) | Treatment  (Cholecalciferol) | 30 | 47±10.65 | 50%  (15/30) | Yes  1.Hypertensio  [9(30%)]  2.Diabetes mellitus  [2(7%)]  3.Diabetes mellitus with complications  [3(10%)]  4.Ischemic cardiomyopathy  [4(13%)]  5.Tobacco use [3(10%)]  6.Peripheral artery disease  [1(3%)] | Yes | Yes  (Not reported in detail) | 17.9 (16.48-20)  ng/ml | 200.000 IU monthly for 3 months； |
|  |  |  |  | Control  (placebo) | 30 | 47.07±12.35 | 53.33%  (16/30) | Yes  1.Hypertensio  [7(23%)]  2.Diabetes mellitus  [3(10%)]  3.Diabetes mellitus with complications  [1(3%)]  4.Ischemic cardiomyopathy  [4(13%)]  5.Tobacco use [2(7%)]  6.Peripheral artery disease  [1(3%)] |  | Yes  (Not reported in detail) | 18.7 (17.3-20.4)  ng/ml | _ |
| Ayub  et al.,2022[18] | RCT | HD | 8w(2m) | Treatment  (Cholecalciferol) | 35 | 49±10.11 | 54.3%  (19/35) | Yes  1.Diabetes  [17(48.6%)]  2.Hepatitis c  [18(51.4%)] | Yes | Yes  (Not reported in detail) | 15.13±5.34  ng/ml | 25(OH)D：  <15 ng/mL→50,000 IU weekly for 2 months；  16–30 ng/mL→10,000 IU weekly for 2 months； |
|  |  |  |  | Control  (placebo) | 35 | 46±14 | 65.7%  (23/35) | Yes  1.Diabetes  [17(48.6%)]  2.Hepatitis c  [17(48.6%)] | Yes | Yes  (Not reported in detail) | 17.03±5.60  ng/ml | _ |
| Gregorio  et al.,2021[19] | RCT | HD | 24w(6m) | Treatment  (Cholecalciferol) | 18 | 59.0  (51.75,70.25) | 50% | Yes  1.Diabetes (50%)  2.Hypertension (25%) | Yes | Yes  1.Calcium  carbonate  (33%)  2.Sevelamer  (67%) | 15.2 (10.6–23.1)  ng/ml | cholecalciferol 50.000 IU weekly for 3month；If after 3 months 25(OH)D was ≥30 ng/ml, 50.000 IU was given monthly; If 25(OH)D was <30 ng/ml, weekly administration was continued； |
|  |  |  |  | Control  (placebo) | 14 | 55.5  (50.50,65.25) | 53% | Yes  1.Diabetes (46.7%)  2.Hypertensio (13.3%) | Yes | Yes  1.Calcium carbonate (29%)  2.Sevelamer  (71%) | 22.0 (17.2–24.1)  ng/ml | _ |
| Marckmann  et al.,2012[20] | RCT | HD | 8w | Treatment  (Cholecalciferol) | 13 | _ | _ | Unable to determine whether to use it or not | Yes | Unable to determine whether to use it or not | 20.7 (16.3–28.9)  nmol/L | 40,000 IU weekly for 8 week； |
|  |  |  |  | Control  (placebo) | 14 | _ | _ | Unable to determine whether to use it or not | Yes | Unable to determine whether to use it or not | 35.9 (25.5–45.9)  nmol/l | _ |
| Miskulin  et al.,2015[21] | RCT | HD | 24w(6m) | Treatment  (Ergocalciferol) | 137 | 61.4±13.3 | _ | Yes  1.Diabetes  [66 (48%)]  2.Hypertensio  [39(28%)] | Yes | Yes  (Not reported in detail,85%) | 16.0±5.9  ng/ml | 25(OH)D：  ≤15 ng/mL→50,000 IU weekly for 6 months；  16–30 ng/mL→50,000 IU 50,000 IU weekly for 3 months，followed by 50,000 IU monthly for 3 months； |
|  |  |  |  | Control  (placebo) | 139 | 60.8±13.9 | _ | Yes  1.Diabetes [61(44%)]  2.Hypertensio [44(32%)] | Yes | Yes  (Not reported in detail,92%) | 16.9±6.4  ng/ml | _ |
| Seibert  et al.,2013[22] | RCT | HD | 12w | Treatment  (Cholecalciferol) | 15 | 66.9±10.8 | 60.0%  (9/15) | Yes  (Not reported in detail) | Yes | Yes  1.Ca-Acetate  [12(4,635 mg/day)]  2.Algeldrate  [4(2,550 mg/day)]  3.Aluminum hydroxide  [2(900 mg/day)]  4.Sevelamer  [4(4,300 mg/day)] | 29.4±11.2  nmol/l | 25(OH)D：  ≤12.5 nmol/L→40,000 IU weekly for 12 weeks；  12.5–37.5 nmol/L→20,000 IU weekly for 12 weeks；  37.5–75 nmol/L→40,000 IU monthly for 12 weeks；  75–150 nmol/L→20,000 IU monthly for 12 weeks； |
|  |  |  |  | Control  (placebo) | 18 | 67.4±9.8 | 50.0%  (9/18) | Yes  (Not reported in detail) | Yes | Yes  1.Ca-Acetate  [7(3,686 mg/day)]  2. Algeldrate  [4 (1,800 mg/day)]  3. Sevelamer  [9 (3,689 mg/day)] | 33.6±16.6  nmol/l | _ |
| Sharif DA.,2022-1/2/3/4[23] | RCT | HD | 8w | Treatment  (Cholecalciferol) | 11/31/6/5 | 59±11.1 | 54.9%  (28/51) | Yes  1.Diabetes [25(49%)]  2.Hypertensio [23(45%)]  3.Atherosclerotic Disease  [16(31%)]  4.Acute Coronary Syndrome  [12(23%)]  5.CV congestion  [5(10%)]  6.Others (smoke)  [23(45%)] | Yes | No relevant description available | 8.61±0.8 ng/ml  12.66±1.6 ng/ml  22.01±2.36 ng/ml  31.25±1.6 ng/ml | The primary dose of 0.25 mg/day for Cholecalciferol was determined on the basis of the levels of plasma calcium and parathyroid hormone (PTH). Dose adjustment for subsequent visits was determined accordingly up to a dose of 0.5 mg daily. |
|  |  |  |  | Control  (placebo) | 10/30/6/5 | 56±15.2 | 60.8%  (31/53) | Yes  1.Diabetes [23(45%)]  2.Hypertensio [27(53%)]  3.Atherosclerotic Disease  [17(33%)]  4.Acute Coronary Syndrome  [13(25%)]  5.CV congestion  [7(14%)]  6.Others (smoke)  [28(49%)] | Yes | No relevant description available | 8.36±0.35 ng/ml  12.70±2.6 ng/ml  21.72±1.3 ng/ml  30.46±1.6 ng/ml | _ |
| Tamadon  et al.,2017[24] | RCT | HD | 12w | Treatment  (Cholecalciferol) | 30 | 60.1±10.4 | 63.4%  (19/30) | Yes  Diabetes (100%) | Yes | Yes  1.Sevelamer  [9(30%)]  2.Calcium carbonate  [21 (70%)] | 15.2±6.7  ng/ml | 50,000 IU every 2 weeks for 12 weeks |
|  |  |  |  | Control  (placebo) | 30 | 65.1±10.1 | 63.4%  (19/30) | Yes  Diabetes (100%) | Yes | Yes  1.Sevelamer  [8(26.7%)]  2.Calcium carbonate  [22(73.3%)] | 15.4±6.9  ng/ml | _ |

Table note: Baseline 25(OH)D levels, dosing regimens, concomitant medications, and phosphate binders are presented as originally reported in the included studies to preserve the original study characteristics and reflect the clinical heterogeneity across trials. For the quantitative analyses, baseline 25(OH)D values were converted and harmonized to ng/mL, using the conversion factor of 1 ng/mL = 2.5 nmol/L. No re-categorization of dosing strategies or concomitant treatments was applied in Table 1.
