# Supplementary material for: The role of nutritional vitamin D on microinflammation and nutritional status in maintenance hemodialysis patients: a meta-analysis of randomized controlled trials
Source: Front Nutr. 2026 May 29;13:1767616. doi: 10.3389/fnut.2026.1767616 (PMC13260404; doi:10.3389/fnut.2026.1767616)
Supplement: Supplementary file 4 [file Supplementary_file_4.docx]

Supplementary File 4

GRADE Evaluation Form

**Comparison:** Vitamin D vs Control
**Population:** MHD
**Follow-up:** 8–24 weeks
**Effect measure:** Continuous outcomes reported as **MD/SMD**; Dichotomous outcomes as **RR**

| **Outcome** | **Type** | **No. of studies (k)** | **Total** | **Effect (95% CI)** | **Certainty of evidence**  **(GRADE)** | **Comments** |
| --- | --- | --- | --- | --- | --- | --- |
| CRP change | Continuous | 7 | 557 | MD = -3.15(-4.46, -1.84) | Low | Downgraded 2 levels for very serious inconsistency (I²=90%) (b) |
| Albumin change | Continuous | 5 | 307 | MD = 0.55(0.10, 1.01) | Low | Downgraded 2 levels for very serious inconsistency (I²=96%) (b) |
| 25(OH)D change | Continuous | 8 | 617 | MD = 15.24(10.27, 20.22) | Low | Downgraded 2 levels for very serious inconsistency (I²=99%) (b) |
| 1,25(OH)₂D change | Continuous | 2 | 58 | MD = 14.50(5.84, 23.16) | Low | Downgraded 2 levels for very serious imprecision (c) |
| Phosphorus change | Continuous | 7 | 557 | MD = 0.55(0.13, 0.97) | Low | Downgraded 2 levels for very serious inconsistency (I²=87%) (b) |
| Calcium change | Continuous | 7 | 557 | MD = 0.07(-0.07, 0.21) | Low | Downgraded 1 level for imprecision (c) and 1 level for suspected publication bias (d). |
| iPTH change | Continuous | 7 | 557 | SMD = -0.56(-1.07, -0.05) | Low | Downgraded 2 levels for very serious inconsistency (I²=84%) (b) |
| ALP change | Continuous | 4 | 220 | SMD = -0.35(-0.83, 0.14) | Low | Downgraded 1 level for inconsistency (I²=61%) (b) and 1 level for imprecision (c). |
| FGF-23 change | Continuous | 4 | 141 | SMD = -0.00(-0.33, 0.33) | Low | Downgraded 2 levels for very serious imprecision (c). |
| TEAEs | Dichotomous | 4 | 368 | RR = 0.86(0.71, 1.05) | Moderate | Downgraded 1 level for imprecision (c). |
| Hypercalcemia incidence | Dichotomous | 3 | 92 | RR = 3.7(0.64, 21.24) | Low | Downgraded 2 levels for very serious imprecision (c). |
| Hyperphosphatemia incidence | Dichotomous | 2 | 65 | RR = 1.73(0.60, 5.02) | Low | Downgraded 2 levels for very serious imprecision (c). |

Table S2. Summary of Effect Estimates for Main Outcomes and Certainty of Evidence Assessment.

a. Risk of bias: downgraded due to concerns regarding methodological limitations in several included trials, including unclear blinding/allocation procedures in some domains.

b. Inconsistency: downgraded due to substantial unexplained heterogeneity across studies; outcomes with very high I² values were downgraded by two levels for very serious inconsistency.

c. Imprecision: downgraded due to small total sample size and/or wide 95% confidence intervals crossing the line of no effect or clinically relevant thresholds.

d. Publication bias: downgraded one level for suspected small-study effects/publication bias in the calcium outcome; this assessment was interpreted cautiously given the limited number of included studies.

| **Outcome** | **Starting**  **level (RCT)** | **Risk of bias** | **Inconsistency** | **Indirectness** | **Imprecision** | **Publication**  **bias** | **Overall**  **certainty** | **Rationale** |
| --- | --- | --- | --- | --- | --- | --- | --- | --- |
| CRP  change | High | No serious | Very serious | No serious | No serious | Undetected | Low | Downgraded 2 levels for very serious inconsistency (I² = 90%). |
| Albumin  change | High | No serious | Very serious | No serious | No serious | Undetected | Low | Downgraded 2 levels for very serious inconsistency (I² = 96%). |
| 25(OH)D  change | High | No serious | Very serious | No serious | No serious | Undetected | Low | Downgraded 2 levels for very serious inconsistency (I² = 99%). |
| 1,25(OH)₂D  change | High | No serious | No serious | No serious | Very serious | Undetected | Low | Downgraded 2 levels for very serious imprecision due to the very small number of studies and participants (2 studies, n = 58). |
| Phosphorus  change | High | No serious | Very serious | No serious | No serious | Undetected | Low | Downgraded 2 levels for very serious inconsistency (I² = 87%). |
| Calcium  change | High | No serious | No serious | No serious | Serious | Suspected | Low | Downgraded 1 level for imprecision because the confidence interval was wide, and 1 level for suspected small-study effects/publication bias. |
| iPTH  change | High | No serious | Very serious | No serious | No serious | Undetected | Low | Downgraded 2 levels for very serious inconsistency (I² = 84%). |
| ALP  change | High | No serious | Serious | No serious | Serious | Undetected | Low | Downgraded 1 level for inconsistency (I² = 61%) and 1 level for imprecision. |
| FGF-23  change | High | No serious | No serious | No serious | Very serious | Undetected | Low | Downgraded 2 levels for very serious imprecision due to limited sample size and uncertainty in the estimate. |
| TEAEs | High | No serious | No serious | No serious | Serious | Undetected | Moderate | Downgraded 1 level for imprecision due to confidence interval including no effect. |
| Hypercalcemia  incidence | High | No serious | No serious | No serious | Very serious | Undetected | Low | Downgraded 2 levels for very serious imprecision due to small total sample size and wide confidence interval. |
| Hyperphosphatemia  incidence | High | No serious | No serious | No serious | Very serious | Undetected | Low | Downgraded 2 levels for very serious imprecision due to very small total sample size and wide confidence interval. |

Table S3. Detailed GRADE Certainty of Evidence Ratings for Each Outcome.

Overall, the certainty of evidence was low for most biochemical outcomes because of substantial heterogeneity and/or imprecision. Certainty was moderate only for TEAEs, while calcium was rated as low certainty because of imprecision and suspected small-study effects/publication bias. These ratings should be interpreted in the context of the limited number of studies and the uncertainty surrounding several pooled estimates.
