# Supplementary material for: The role of nutritional vitamin D on microinflammation and nutritional status in maintenance hemodialysis patients: a meta-analysis of randomized controlled trials
Source: Front Nutr. 2026 May 29;13:1767616. doi: 10.3389/fnut.2026.1767616 (PMC13260404; doi:10.3389/fnut.2026.1767616)
Supplement: Supplementary file 5 [file Supplementary_file_5.docx]

Supplementary File 5

**Sensitivity analysis using the Hartung-Knapp-Sidik-Jonkman adjustment**

The Hartung-Knapp-Sidik-Jonkman adjustment was applied to the random-effects meta-analysis using the same study-level effect estimates and standard errors extracted from the RevMan file. To maintain consistency with the original RevMan random-effects analyses, the between-study variance was estimated using the DerSimonian-Laird method. The pooled effect estimates remained unchanged, whereas the 95% confidence intervals were recalculated using the HKSJ adjustment.

For continuous outcomes, mean difference or standardized mean difference was used according to the original analysis. For dichotomous safety outcomes, risk ratio was used. The HKSJ-adjusted results were compared with the conventional random-effects results to evaluate whether the statistical significance and interpretation of each outcome changed.

The HKSJ-adjusted analysis was conducted only as a supplementary sensitivity analysis and was not used as the primary basis for interpreting the study findings. The main results and conclusions of this study were based on the conventional random-effects meta-analysis, while the HKSJ-adjusted estimates and corresponding forest plots were provided as supplementary materials to evaluate the robustness of the results.

**Overall findings**

Overall, the HKSJ-adjusted analyses yielded pooled point estimates that were generally consistent with the conventional random-effects analyses. However, the 95% confidence intervals became wider after HKSJ adjustment, reflecting a more conservative estimation of uncertainty.

The findings for CRP, 25(OH)D, 1,25(OH)₂D, and phosphate remained statistically significant after HKSJ adjustment. In contrast, the initially significant findings for albumin and iPTH were no longer statistically significant after HKSJ adjustment. Calcium, ALP, FGF-23, TEAEs, hyperphosphatemia, TNF-α, IL-1β, and WBC remained non-significant in both the conventional and HKSJ-adjusted analyses. Hypercalcemia became statistically significant after HKSJ adjustment, but this result should be interpreted cautiously because it was based on a small number of studies and sparse event data.

| **Outcome** | **Conventional random-effects model** | **HKSJ-adjusted random-effects model** | **Interpretation** |
| --- | --- | --- | --- |
| CRP change | MD = −3.15 [−4.46, −1.84] | MD = −3.15 [−5.22, −1.08] | Remained significant |
| CRP change,vitamin D deficiency subgroup | MD = −2.64 [−4.47, −0.81] | MD = −2.64 [−5.61, 0.33] | No longer significant |
| CRP change, normal vitamin D subgroup | MD = −3.97 [−5.78, −2.17] | MD = −3.97 [−15.70, 7.75] | No longer significant |
| CRP change, duration ≤12 weeks | MD = −3.80 [−5.07, −2.53] | MD = −3.80 [−5.98, −1.62] | Remained significant |
| CRP change, duration >12 weeks | MD = 0.17 [−1.70, 2.04] | MD = 0.17 [−1.85, 2.18] | Remained non-significant |
| Albumin change | MD = 0.55 [0.10, 1.01] | MD = 0.55 [−0.01, 1.11] | No longer significant |
| 25(OH)D change | MD = 15.24 [10.27, 20.22] | MD = 15.24 [6.36, 24.13] | Remained significant |
| 1,25(OH)₂D change | MD = 14.50 [5.84, 23.16] | MD = 14.50 [5.08, 23.92] | Remained significant |
| Phosphate change | MD = 0.55 [0.13, 0.97] | MD = 0.55 [0.08, 1.02] | Remained significant |
| Calcium change | MD = 0.20 [−0.05, 0.44] | MD = 0.20 [−0.12, 0.52] | Remained non-significant |
| iPTH change | SMD = −0.56 [−1.07, −0.05] | SMD = −0.56 [−1.16, 0.03] | No longer significant |
| ALP change | SMD = −0.35 [−0.83, 0.14] | SMD = −0.35 [−0.98, 0.28] | Remained non-significant |
| FGF-23 change | SMD = −0.00 [−0.33, 0.33] | SMD = −0.00 [−0.41, 0.40] | Remained non-significant |
| TEAEs | RR = 0.84 [0.69, 1.03] | RR = 0.84 [0.64, 1.12] | Remained non-significant |
| Hypercalcemia | RR = 3.62 [0.62, 21.25] | RR = 3.62 [1.29, 10.20] | Became significant |
| Hyperphosphatemia | RR = 1.71 [0.59, 4.93] | RR = 1.71 [0.18, 16.29] | Remained non-significant |
| TNF-α change | MD = −0.65 [−1.38, 0.07] | MD = −0.65 [−1.55, 0.25] | Remained non-significant |
| IL-1β change | SMD = −0.42 [−1.50, 0.65] | SMD = −0.42 [−2.16, 1.32] | Remained non-significant |
| WBC change | MD = −0.43 [−1.30, 0.43] | MD = −0.43 [−2.67, 1.80] | Remained non-significant |

Abbreviations: HKSJ, Hartung-Knapp-Sidik-Jonkman; MD, mean difference; SMD, standardized mean difference; RR, risk ratio; CI, confidence interval; CRP, C-reactive protein; iPTH, intact parathyroid hormone; ALP, alkaline phosphatase; FGF-23, fibroblast growth factor 23; TEAEs, treatment-emergent adverse events; WBC, white blood cell count.

**Results by outcome**

**CRP change**

For CRP change, the conventional random-effects model showed that vitamin D supplementation was associated with a significant reduction in CRP levels compared with control, with a pooled mean difference of −3.15 and a 95% CI of −4.46 to −1.84. After HKSJ adjustment, the pooled estimate remained unchanged, while the confidence interval widened to −5.22 to −1.08. The overall effect therefore remained statistically significant, suggesting that the main CRP result was robust to HKSJ adjustment.

In the subgroup analysis based on baseline vitamin D status, the confidence intervals widened after HKSJ adjustment. In the vitamin D deficiency subgroup, the result changed from MD = −2.64 [−4.47, −0.81] to MD = −2.64 [−5.61, 0.33]. In the normal vitamin D subgroup, the result changed from MD = −3.97 [−5.78, −2.17] to MD = −3.97 [−15.70, 7.75]. Thus, the subgroup-specific CRP findings were no longer statistically significant after HKSJ adjustment and should be interpreted cautiously.

In the subgroup analysis based on treatment duration, the CRP-lowering effect remained statistically significant in studies with a duration of 12 weeks or less, changing from MD = −3.80 [−5.07, −2.53] to MD = −3.80 [−5.98, −1.62] after HKSJ adjustment. In contrast, the subgroup with treatment duration longer than 12 weeks remained non-significant, changing from MD = 0.17 [−1.70, 2.04] to MD = 0.17 [−1.85, 2.18]. These findings suggest that the CRP-lowering effect was mainly driven by studies with shorter treatment duration.

**Albumin change**

For albumin change, the conventional random-effects model suggested a statistically significant increase in albumin levels, with MD = 0.55 [0.10, 1.01]. After HKSJ adjustment, the confidence interval widened and crossed the null value, with MD = 0.55 [−0.01, 1.11]. Therefore, the statistical significance of albumin change was not retained after HKSJ adjustment, and this finding should be interpreted with caution.

**25(OH)D change**

For 25(OH)D change, the conventional random-effects model showed a significant increase in 25(OH)D levels, with MD = 15.24 [10.27, 20.22]. After HKSJ adjustment, the pooled estimate remained unchanged and the confidence interval remained entirely above zero, with MD = 15.24 [6.36, 24.13]. This indicates that the effect of vitamin D supplementation on increasing 25(OH)D levels was robust to HKSJ adjustment.

**1,25(OH)₂D change**

For 1,25(OH)₂D change, the conventional random-effects model showed a significant increase, with MD = 14.50 [5.84, 23.16]. After HKSJ adjustment, the result remained statistically significant, with MD = 14.50 [5.08, 23.92]. However, because this analysis included only a small number of studies, the result should still be interpreted with appropriate caution.

**Phosphate change**

For phosphate change, the conventional random-effects model showed a significant increase in phosphate levels, with MD = 0.55 [0.13, 0.97]. After HKSJ adjustment, the confidence interval became wider but remained above zero, with MD = 0.55 [0.08, 1.02]. Therefore, the phosphate result remained statistically significant after HKSJ adjustment.

**Calcium change**

For calcium change, the conventional random-effects model did not show a statistically significant difference between groups, with MD = 0.20 [−0.05, 0.44]. After HKSJ adjustment, the confidence interval widened to MD = 0.20 [−0.12, 0.52], and the result remained non-significant. This suggests that there was no robust evidence of a treatment effect on calcium levels.

**iPTH change**

For iPTH change, the conventional random-effects model suggested a statistically significant reduction, with SMD = −0.56 [−1.07, −0.05]. After HKSJ adjustment, the confidence interval widened and crossed the null value, with SMD = −0.56 [−1.16, 0.03]. Therefore, the statistical significance of the iPTH result was not retained after HKSJ adjustment.

**ALP change**

For ALP change, the conventional random-effects model showed no statistically significant difference between groups, with SMD = −0.35 [−0.83, 0.14]. After HKSJ adjustment, the confidence interval widened to SMD = −0.35 [−0.98, 0.28], and the result remained non-significant.

**FGF-23 change**

For FGF-23 change, the conventional random-effects model showed no statistically significant difference between groups, with SMD = −0.00 [−0.33, 0.33]. After HKSJ adjustment, the confidence interval widened slightly to SMD = −0.00 [−0.41, 0.40], and the result remained non-significant.

**Treatment-emergent adverse events**

For treatment-emergent adverse events, the conventional random-effects model showed no statistically significant difference between groups, with RR = 0.84 [0.69, 1.03]. After HKSJ adjustment, the result remained non-significant, with RR = 0.84 [0.64, 1.12]. This suggests that vitamin D supplementation was not associated with a statistically significant difference in overall treatment-emergent adverse events.

**Hypercalcemia**

For hypercalcemia, the conventional random-effects model showed no statistically significant difference between groups, with RR = 3.62 [0.62, 21.25]. After HKSJ adjustment, the confidence interval changed to RR = 3.62 [1.29, 10.20], suggesting a statistically significant increase. However, this finding should be interpreted cautiously because the analysis was based on only three studies and very few events, with no events observed in the control groups.

**Hyperphosphatemia**

For hyperphosphatemia, the conventional random-effects model showed no statistically significant difference between groups, with RR = 1.71 [0.59, 4.93]. After HKSJ adjustment, the confidence interval became much wider, with RR = 1.71 [0.18, 16.29], and the result remained non-significant. This indicates substantial uncertainty around the pooled estimate.

**TNF-α change**

For TNF-α change, the conventional random-effects model showed no statistically significant difference between groups, with MD = −0.65 [−1.38, 0.07]. After HKSJ adjustment, the confidence interval widened to MD = −0.65 [−1.55, 0.25], and the result remained non-significant.

**IL-1β change**

For IL-1β change, the conventional random-effects model showed no statistically significant difference between groups, with SMD = −0.42 [−1.50, 0.65]. After HKSJ adjustment, the confidence interval widened markedly to SMD = −0.42 [−2.16, 1.32], and the result remained non-significant. This indicates considerable uncertainty around the pooled estimate.

**WBC change**

For WBC change, the conventional random-effects model showed no statistically significant difference between groups, with MD = −0.43 [−1.30, 0.43]. After HKSJ adjustment, the confidence interval widened to MD = −0.43 [−2.67, 1.80], and the result remained non-significant.

**Conclusion**

The HKSJ-adjusted sensitivity analyses generally supported the robustness of the main findings for CRP, 25(OH)D, 1,25(OH)₂D, and phosphate. However, the statistical significance of albumin and iPTH was not retained after HKSJ adjustment, suggesting that these outcomes should be interpreted cautiously. Most safety outcomes remained non-significant after HKSJ adjustment, including TEAEs and hyperphosphatemia. Hypercalcemia became statistically significant after HKSJ adjustment, but this finding was based on sparse event data and a small number of studies; therefore, it should be considered exploratory and interpreted with caution.

Overall, the HKSJ adjustment did not materially change the direction of the pooled estimates, but it widened the confidence intervals and reduced the certainty of several findings. These results indicate that the primary conclusions were broadly consistent; nevertheless, additional well-designed studies with larger sample sizes and more complete outcome data are warranted to further validate these findings and support more detailed analyses.
